# Citrate: a key signalling molecule and therapeutic target for bone remodeling disorder

**DOI:** 10.3389/fendo.2024.1512398

**Published:** 2025-01-16

**Authors:** Qichang Liu, Yuchuan Xue, Junzhe Guo, Lin Tao, Yue Zhu

**Affiliations:** Department of Orthopedics, The First Hospital of China Medical University, Shenyang, China

**Keywords:** osteoporosis, bone remodeling, energy metabolism, citrate, osteoblast

## Abstract

Bone remodeling is a continuous cyclic process that maintains and regulates bone structure and strength. The disturbance of bone remodeling leads to a series of bone metabolic diseases. Recent studies have shown that citrate, an intermediate metabolite of the tricarboxylic acid (TCA) cycle, plays an important role in bone remodeling. But the exact mechanism is still unclear. In this study, we focused on the systemic regulatory mechanism of citrate on bone remodeling, and found that citrate is involved in bone remodeling in multiple ways. The participation of citrate in oxidative phosphorylation (OXPHOS) facilitates the generation of ATP, thereby providing substantial energy for bone formation and resorption. Osteoclast-mediated bone resorption releases citrate from bone mineral salts, which is subsequently released as an energy source to activate the osteogenic differentiation of stem cells. Finally, the differentiated osteoblasts secrete into the bone matrix and participate in bone mineral salts formation. As a substrate of histone acetylation, citrate regulates the expression of genes related to bone formation and bone reabsorption. Citrate is also a key intermediate in the metabolism and synthesis of glucose, fatty acids and amino acids, which are three major nutrients in the organism. Citrate can also be used as a biomarker to monitor bone mass transformation and plays an important role in the diagnosis and therapeutic evaluation of bone remodeling disorders. Citrate imbalance due to citrate transporter could result in the supression of osteoblast/OC function through histone acetylation, thereby contributing to disorders in bone remodeling. Therefore, designing drugs targeting citrate-related proteins to regulate bone citrate content provides a new direction for the drug treatment of diseases related to bone remodeling disorders.

## 1 Introduction

### 1.1 Disorder of bone remodeling and skeletal conditions

Bone remodeling is a continuous process that regulates the mass and strength of bones. By means of this process, old bone is substituted by new bone. In addition to supporting bone growth, bone remodeling also functions as a reparative mechanism for damaged bones following the rule of fractures and micro-injuries (1). Bone remodeling involves the coordinated activity of osteoblasts and osteoclasts (2). Bone tissue undergoes constant remodeling. The imbalances in this process are closely associated with various skeletal disorders (3), such as osteoporosis, a prevalent degenerative bone disease characterized by decreased bone quality and increased turnover resulting from an imbalance between resorption and replacement during remodeling (4). Other skeletal disorders, such as osteoarthritis and rheumatoid arthritis, involve disruptions in both cartilage resorption and formation. Cancer-associated bone diseases can lead to calcium loss in bones and diffuse absorption due to hypercalcemia (5). Therefore, targeting healthy and diseased bones through remodeling behavior represents the fundamental approach to prevent and treating bone diseases (6).

### 1.2 Many factors systematically regulate bone remodeling

Many factors systematically regulate bone remodeling. The equilibrium of bone remodeling is dynamic and influenced by numerous factors, including ATP as the core component of energy supply, post-translational modification of proteins, metabolic programming, immune and inflammatory factors, among others indicating that the homeostasis of bone remodeling is not only influenced by bone resorption but also by systemic factors. ATP plays a crucial role in bone remodeling as it serves as the central component for the energy supply. Osteoblasts are essential in creating new bone mass and enhancing bone density throughout the processes of bone growth, development, and continuous remodeling; these processes require a substantial amount of energy to which ATP significantly contributes (7). Osteoclasts are responsible for bone resorption and also need to generate significant quantities of ATP through glycolysis and oxidative phosphorylation (OXPHOS) (8). Studies have indicated impaired key enzymes for ATP production in patients with osteoporosis (9), suggesting that disrupted ATP-facilitated energy provision plays a crucial part in disturbed bone remodeling. Metabolic reprogramming also plays an important role in disorders related to bone remodeling. The metabolic reprogramming of osteoblasts and osteoclasts has emerged as a critical approach for promoting bone regeneration and managing osteoporosis (10, 11). Bone remodeling is significantly influenced by the immune system. Inflammation in the bone leads to a higher rate of bone absorption compared to formation, resulting in overall bone loss (inflammation-induced osteolysis) (12). The immune system primarily relies on immune-inflammatory factors to regulate bone remodeling; therefore, targeting these factors is crucial for treating disorders related to bone remodeling. Additionally, histone modification also plays a critical role in the process of bone remodeling. Research has demonstrated that abnormalities in histone modification are significant contributors to this process (13), and targeting histone modification has become an important approach for treating osteoporosis (14). In conclusion, multiple factors systematically regulate bone remodeling. The fundamental solution lies in exploring the systemic pathogenesis of bone disorders, identifying key nodes within the system’s dysfunctions, and implementing targeted treatments.

### 1.3 Citrate and bone remodeling

Among the numerous metabolic products, citrate is a very unique metabolite. On the one hand, it can enter the TCA cycle to participate in its functions; on the other hand, during this process, the cleavage of citrate produces a large amount of ace-CoA, which serves as the sole substrate for histone acetylation modification (15). Therefore, citrate may be considered an essential metabolite involved in the multi-level regulation of bone remodeling. Citrate serves as a crucial substrate for cellular energy metabolism, being generated in the mitochondria and utilized in the Krebs cycle or transported into the cytoplasm by the dedicated mitochondrial carrier CIC(mitochondrial citrate transporter). Within the cytoplasm, citrate and its derivatives, such as acetyl-CoA and oxaloacetate, are implicated in both regular and pathological processes (16). In addition to its role as an energy regulator, citrate also plays diverse roles including maintenance of protein acetylation, lipid synthesis and breakdown, amino acid production, and immune responses (17). Firstly, citrate acts as a key metabolite in the TCA cycle to maintain OXPHOS, ultimately leading to ATP production through complex V or ATP synthase (18). Moreover, citrate derivatives like acetyl-CoA serve as substrates for acetylation modifications that significantly contribute to histone acetylation. Additionally, citrate is believed to play a crucial role in metabolic reprogramming under various physiological and pathological conditions such as inflammation, Behcet’s syndrome, and heart development (19–21), while also modulating the release of inflammatory factors by the immune system (22). Abnormal levels of citrate can lead to an imbalance in bone remodeling, resulting in bone metabolism-related diseases, which further underscores the importance of citrate in the process of bone remodeling. In conclusion, citrate is now recognized not only as a solitary energy metabolite but also as a pivotal component contributing to systemic homeostasis. Furthermore, citrate holds significance in regulating bone remodeling with disrupted levels observed in bone tissue and plasma of individuals with osteoporosis (23). Notably, citrate has demonstrated efficacy against osteoporosis (24). However, the mechanisms underlying how citrate regulates bone remodeling along with therapeutic effects on bone metabolic disorders remain unclear.

In this review, we will explore the role of citrate in bone remodeling, elucidate its molecular mechanisms, and discuss its potential clinical applications. This review will introduce the innovative concept that citrate regulates bone remodeling by influencing ATP energy supply, histone acetylation, metabolic programming, and immune-inflammatory factors.

## 2 Citrate in the circulation of the human system

**Figure 1:**
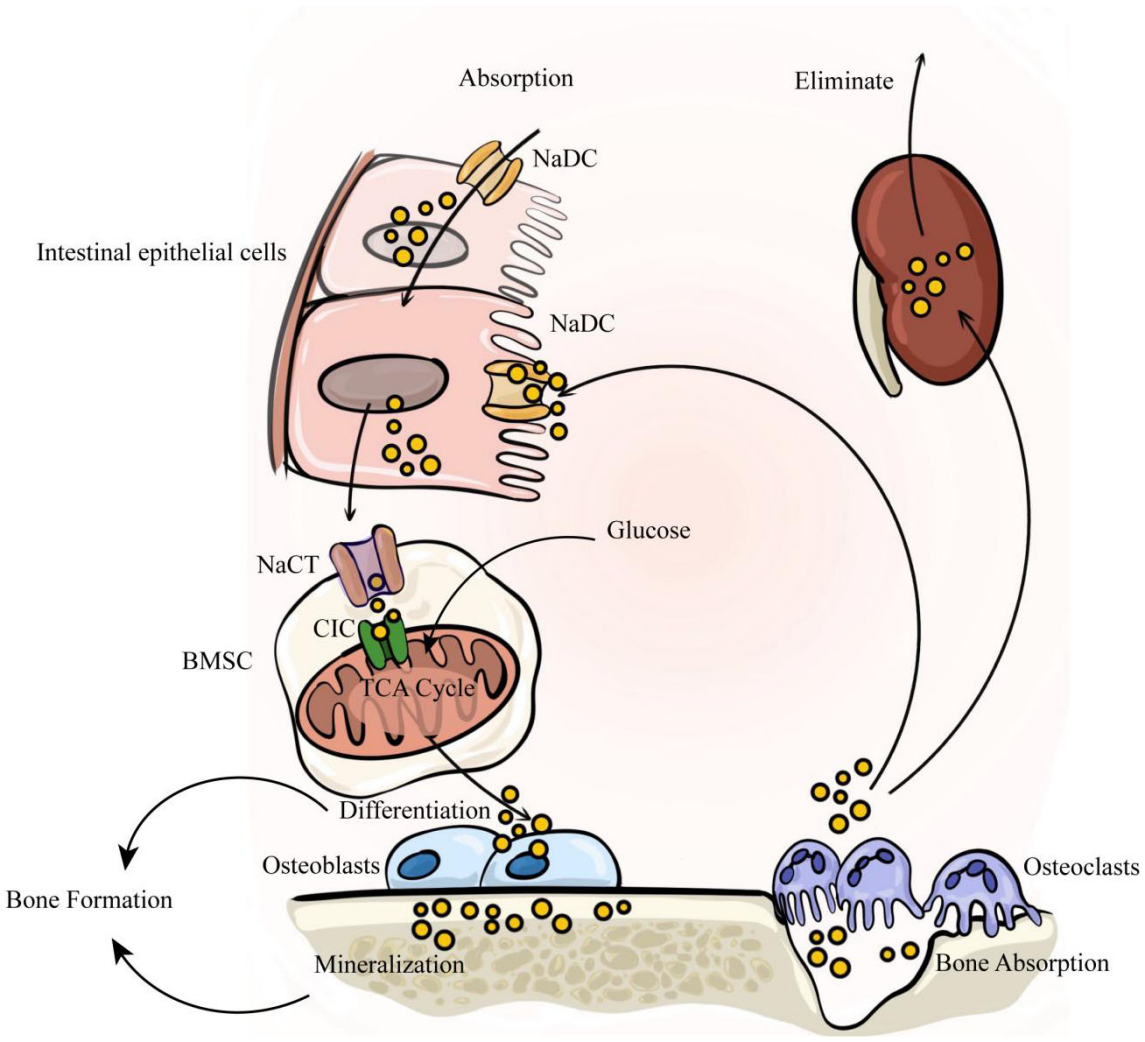
The internal circulation of citrate relies on four factors: nutrient intake, renal clearance, cellular metabolism, and bone remodeling. Citrate is primarily absorbed through dietary intake, enters the bloodstream via intestinal absorption, and is subsequently eliminated by the kidneys to maintain a dynamic balance of citrate in the body. Intestinal absorption and renal reabsorption depend on NaDC. At the same time, citrate within bone tissue serves as a crucial component of bone mineralization. It is stored in the bone matrix and released during osteoclast-mediated bone resorption. The released citrate enters the blood circulation, with a portion being taken up by bone marrow stromal cells via NaCT to activate osteogenic differentiation of stem cells. Subsequently, osteoblasts secrete citrate to participate in new bone formation.

The balance between availability and elimination of citrate is maintained by physiological requirements. Citrate in the human body exists as citrate ions and solid salts. Citrate ions are primarily generated through two pathways: direct ingestion via the gastrointestinal tract and cellular production through the metabolism of various energy substances. Solid citrate is predominantly stored in mineralized tissues, including bones and teeth. Renal metabolism plays a crucial role in the clearance of citrate to uphold body citrate homeostasis. In summary, nutrient intake, renal clearance, cellular metabolism, and bone remodeling collectively determine citrate homeostasis (**Figure 1**).

### 2.1 Citrate intake and clearance

The sources of citrate intake are diverse, including citrus fruits, food and beverage additives, etc. Industrial-grade citrate serves various functions such as preventing food spoilage and regulating acidity (25–27). The typical daily nutritional intake of citrate is approximately 4 grams (28). Over 95% of citrate is absorbed by the small intestinal epithelial cells through the Na+dependent dicarboxylate cotransporter (NaDC). The rapidly increasing levels of citrate in plasma are promptly filtered by the kidneys, which also rely on NaDC (27, 29–31). Around 99% of plasma citrate exists as a tricarboxylate or dicarboxylate complexed with divalent ions like calcium and magnesium. In summary, the balance of plasma citrate depends on its intake through the small intestine and renal clearance, with an important role played by NaDC. However, studies have shown that the intake and clearance of citrate salts cannot fully maintain stable plasma concentrations. Therefore, it is necessary to identify factors affecting the stability of plasma citrate concentrations. Abundant amounts of citrate are also found in cells and bone tissue (30, 31).

### 2.2 The citrate cycle in cells

Cellular metabolism has minimal impact on citrate availability, as it is predominantly sequestered within the mitochondria and not exchanged with the extracellular space. Citrate is synthesized in the mitochondrial matrix through the enzymatic action of citrate synthase (CS), which catalyzes the condensation of acetyl-CoA and oxaloacetate to form citrate. This generated citrate then enters the TCA cycle to provide cellular energy. To proceed further in the Krebs cycle, citrate undergoes isomerization to isocitrate facilitated by aconitase (ACO2). Abundant mitochondrial citrate can be transported to the cytosol via the mitochondrial CIC. In the cytosol, ACLY (ATP citrate lyase) cleaves citrate into acetyl-CoA and oxaloacetate. A portion of acetyl-CoA is subsequently carboxylated into malonyl-CoA, which participates directly in lipid metabolism by condensing into long-chain fatty acids. The remaining acetyl-CoA serves as a substrate for histone acetylation and provides acetyl groups for protein modification under the influence of acetyltransferase enzymes. Furthermore, intracellular citrate also regulates glycolysis, gluconeogenesis, and fatty acid oxidation (16). Only select cell types absorb or release extracellular citrate due to various physiological reasons (17), such as osteocytes secreting citrates involved in bone mineralization or intestinal epithelial cells and renal tubules absorbing extracellular citrates through NaDC family transporters.

### 2.3 Citrate in bone tissue

Studies indicate that approximately 90% of citrate in the human body is stored in mineralized tissues, playing a crucial role in regulating metabolic functions and maintaining the structural integrity of bones (32). The primary constituents of bone tissue consist of both organic and inorganic substances. The inorganic component, referred to as bone salts, primarily comprises hydroxyapatite, which aligns along the elongated axis of collagen fibers and contains a high concentration of calcium and phosphorus (33). Osteoblasts synthesize and secrete the organic portion, which includes around 10% amorphous bone matrix and approximately 90% collagen. Collagen forms a gel-like substance rich in glycine, alanine, proline, and hydroxyproline with neutral or weakly acidic glycosaminoglycans predominantly composed of type I collagen alongside a small amount of type V collagen. The amorphous bone matrix mainly consists of proteoglycans, polysaccharide complexes, as well as osteocalcin-like osteonectin (34). Within the organic component lies about 1-5% citrate content while over 15% surface area of apatite within bones is occupied by citrate molecules (35). These findings suggest that citrate not only plays an essential role in cellular metabolism but also actively participates in vital processes associated with bone matrix development and mineralization.

## 3 Citrate regulates bone mineralization

The citrate-hydroxyapatite combination serves as the fundamental constituent of bone mineral salt, with approximately 70% of bone mass primarily composed of nano-scale citrate-hydroxyapatite crystals measuring 5 × 25 × 50 nm in diameter, exhibiting a delicate plate-like structure (36). Citrate plays a crucial role in the formation of plate-like structures within bone crystals and is tightly bound to hydroxyapatite as an integral component (37). This suggests that citrate cannot be substituted in the process of apatite-based bone tissue formation.

In general, citrate plays four major roles in bone mineralization: it slows down the deposition of calcium and phosphate, promotes stable nucleation of calcium phosphate(CaP), maintains the lamellar growth of apatite, and limits excessive formation of apatite crystals to maintain optimal mechanical conditions. The initiation of bone mineralization begins with the creation of amorphous CaP within highly saturated solutions containing CaP (38). During the initial phases, a limited number of citrate molecules can adhere to the surface of small amorphous CaP clusters, effectively impeding particle aggregation. Research has demonstrated that citrate inhibits hydroxyapatite nucleation by interacting with calcium ions and attaching to crystal surfaces (39, 40). Recent studies suggest that citrate’s capacity to stabilize CaP formations is essential in postponing the emergence of liquid or solid phases required for the formation of the CaP liquid precursor phase. Furthermore, by stabilizing early CaP precursors including nucleating precursor material and the liquid precursor phase, citrate significantly delays solid CaP nucleation (41). This suggests that citrate plays a significant role in the formation of stable CaP precursor materials with appropriate dimensions during initial bone mineralization. In the subsequent stage, nucleation of CaP precursors occurs and non-collagenous proteins secreted by bone cells guide the attachment of CaP to collagen surfaces (42), while citrate facilitates plate-like arrangement of CaP for normal bone tissue formation (43). During the final stage, citrate salts completely envelop the surface of CaP crystals, resulting in unique geometric shapes of hydroxyapatite and forming nanocrystals as mentioned earlier. Citrate coverage prevents further crystal growth and contributes to optimal mechanical structure formation in bone tissue (43).

Citrate has been used to explain changes observed in various bone diseases. Sodium-coupled citrate transporter (NaCT) is responsible for extracellular-to-cell transport of citrate. Research indicates that *SLC13A5*(the gene encoding NaCT) deficiency leads to decreased BMD and impaired bone formation in homozygous and heterozygous knockout mice (44). A subsequent study revealed that mice lacking *SLC13A5* exhibited structural and biomechanical properties indicative of abnormal mineralization. Additionally, the researchers observed excessive citrate accumulation in the bones of *SLC13A5* deficient mice, which may have contributed to reduced cortical thickness and impaired cortical strength (45). This could be attributed to citrate deposition affecting hydroxyapatite formation and decreased citrate coverage impacting water-mineral integrity bound to the bone surface. In brief, Citrate serves as a vital component of hydroxyapatite, where its incorporation into bone minerals and spatial arrangement within the mineral structure is indispensable for preserving the biomechanical properties of bone, including stability, strength, and fracture resistance.

## 4 Citrate circulates during bone remodeling

As previously mentioned, bone remodeling represents the dynamic balance between bone formation and resorption in mature bone tissue, with citrate playing a crucial role in mediating this interplay (**Figure 1**). Recently, research has shown that osteoblasts are the primary origin of citrate in bone tissue. A study by Costello et al. (2012) revealed the secretion of citrate by osteoblasts in mice (46). While calcium citrate acts as an intermediary for calcium exchange between bone and blood, the exact origin of plasma citrate remains uncertain. Experiments utilizing C13 isotope-labeled glucose tracers have suggested that mitochondrial citrate derived from glucose deposition occurs during the later stages of osteogenic differentiation in bone marrow mesenchymal stem cells (BMSC) (47) Interestingly, undifferentiated BMSCs do not possess the capacity to secrete citrate (48). This suggests that citrate found in bone is derived from differentiated BMSCs rather than plasma citrate. BMSCs have the ability to differentiate into various cell types, including osteoblasts within bone tissue (49). Research indicates that osteoblasts are specifically responsible for producing citrate in bone tissue (50), highlighting the significant role of citrate in osteogenic differentiation. *In vitro* studies demonstrate an increase in citrate production with osteoblast differentiation, accompanied by changes in protein expression related to citrate secretion. The deposition of citrate into the bone matrix relies on the net production of citrate by osteoblasts and involves molecular activities such as: Citrate synthetase CS, mitochondrial aconitase (m-acon, which converts citrate to isocitrate), CIC and NaCT. Citrates produced by osteoblasts during mineralization are subsequently released upon breakdown by osteoclasts. Osteoclasts originate from monocyte-macrophages and play a crucial role in degrading the bone matrix. They are formed through fusion of monocytic precursors belonging to monocyte/macrophage lineage and serve as primary resorptive cells within bones (51, 52) Mature osteoclasts adhere to the bone surface through αvβ integrin, establishing an F-actin sealing zone for efficient absorption of the bone matrix. The acidic microenvironment in the resorption area is generated by carbonic anhydrase II, which produces HCO and H ions that are transported by vacuolar H adenosine triphosphatase (V-ATPase) within the folded structure of osteoclasts (53, 54). In this acidic environment of the resorption lacuna, inorganic minerals dissolve while exposing the organic collagen matrix to enzymatic degradation facilitated by proteolytic enzymes such as collagenase, cathepsin K (CTSK), and matrix metalloproteinases (MMP) (54, 55). Subsequently, these enzymes proceed with degrading the exposed collagen and other organic components (56–58), meanwhile, citrate is also released during this process (59). To summarize, within intact bone tissue, osteoblasts secrete citrate to assist in bone matrix formation whereas osteoclasts break down released citrate from the bone matrix, which further provides energy for stem cells and supports their differentiation.

## 5 Citrate as a fuel for bone remodeling

**Figure 2:**
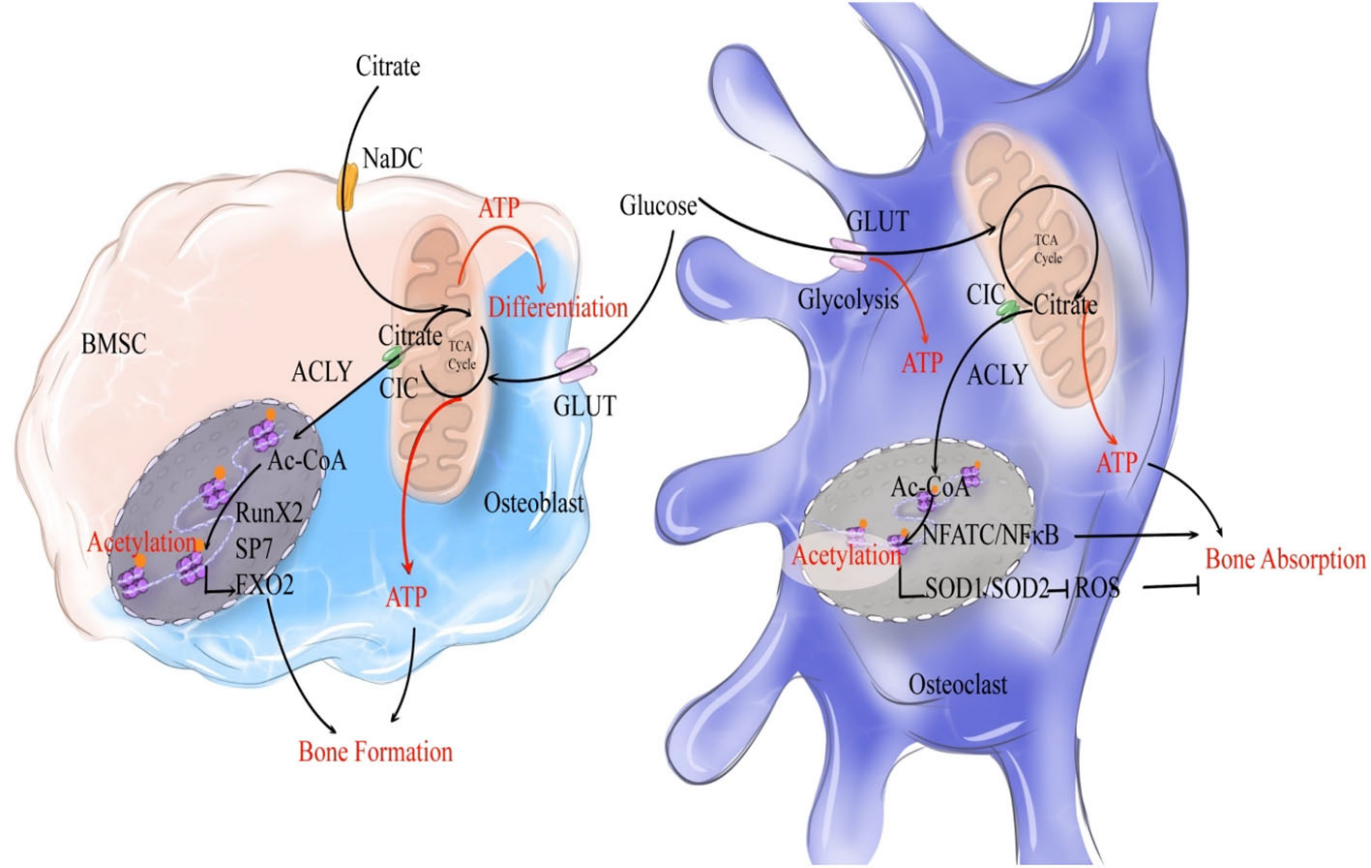
Citrate regulates bone remodeling through ATP production and histone acetylation. During the process of bone formation, NaCT-mediated uptake of exogenous citrate by bone marrow stromal cells (BMSCs, indicated by a light pink cell) activates their differentiation into osteoblasts. Exogenous citrate serves a dual role: it generates ATP via TCA cycle to fuel osteoblastic differentiation, while also being cleaved into acetyl-CoA to enhance the expression of osteogenic genes. Once activated and differentiated into osteoblasts (indicated by a light blue cell), glucose metabolism becomes the primary source of citrate production, as glucose undergoes OXPHOS to generate citrate that provides both ATP and acetyl-CoA for promoting osteogenic gene expression and subsequent acetylation. In terms of bone resorption, glucose metabolism produces citrate that supplies ATP for osteoclastic bone resorption. Moreover, citrate enhances acetylation to stimulate NFATC/NFκB expression and induce osteoclast (indicated by a dark blue cell) differentiation. Simultaneously, it promotes oxidase expression to inhibit the pro-resorptive effect of ROS on bones. Overall, citrate plays a crucial role in promoting bone remodeling.

As mentioned above, citrate circulates among osteoblasts and osteoclasts during bone remodeling to participate in the formation of bone matrix and osteoblastic differentiation. During this process, bone formation is highly energy-consuming, necessitating the substantial production of ATP. Meanwhile, the differentiation process of osteoclasts requires the rearrangement of the cytoskeleton from mononuclear to multinuclear cells and cell fusion, which also necessitates a significant amount of ATP (60). Moreover, the migration of osteoclasts along the bone surface facilitates ongoing bone resorption, involving dynamic rearrangements of the actin and microtubule cytoskeleton, which necessitates significant ATP consumption (61, 62). The TCA cycle is essential for supplying the energy needed for numerous cellular functions, which requires the involvement of cirtrate (63). Abnormal ATP synthesis could result in imbalances in bone metabolism (64–66). Therefore, in addition to its role in bone mineralization, citrate can enter the TCA cycle to provide a substantial amount of ATP, suggesting that citrate may act as a fuel in the process of bone remodeling (**Figure 2**).

### 5.1 Citrate participates in TCA cycle to produce substantial ATP

In the TCA cycle, citrate plays a crucial role in sustaining reduced FADH2 and NADH (nicotinamide adenine dinucleotide) levels, which are utilized in the electron transport chain for oxidative phosphorylation and ATP production through complex V (67). Numerous proteins contribute to the regulation of citrate-mediated ATP, and disturbances in these associated proteins can lead to disorders in osteogenesis and osteoclast function, resulting in various diseases.

### 5.2 Citrate as a fuel in bone remodeling

During this process, citrate acts as a key intermediate product in the energy supply and plays a critical role as an energy source for bone tissue. Upon initiation of osteogenic differentiation, there is an increased demand for metabolic energy in MSCs leading to elevated production of citrate regulated by various enzymes related to citrate metabolism (68).

#### 5.2.1 Extracellular citrate as a fuel in early stage of bone formation

Research has demonstrated that extracellular citrate significantly influences the osteogenic differentiation of MSCs, confirming its role as a fuel for stem cell differentiation (69). This “metabolic” regulation begins with the absorption of citrate through NaCT, activating the energy production pathway that enhances cellular energy status. Consequently, this facilitates meeting high metabolic demands during MSC differentiation into osteoblasts. Notably, this effect appears to be both time- and dose-dependent; however, the dose-dependent impact is more significant during early stages of osteogenesis (69). Additionally, ATP generated from exogenous citrate was found to promote the early stages of osteogenic differentiation (70, 71).

However, the expression and functionality of NaCT following the differentiation of MSCs into osteoblasts remain controversial, suggesting that the demand for extracellular citrate may decrease during the later stages of osteoblastic differentiation. In contrast to the significant expression of various types of transporters in HepG2 cells, Costello et al. found that NaCT transporters are absent in mature osteoblasts (50). On the contrary, studies have demonstrated the presence of NaCT mRNA in bone and primary osteoblasts. Furthermore, reduced bone mineral density and impaired bone formation have been linked to diminished expression of *SLC13A5* which codes NaCT in 13-week-old mice, as well as overall growth retardation, shortened body length, and reduced bone size (44). Additionally, Mantila Roosa SM et al.’s study revealed that local mechanical loading stimulation can upregulate bone NaCT expression (72), thereby increasing bone tissue content. In summary, during early stages of osteogenic differentiation, exogenous citrate intake provides a substantial amount of ATP for BMSCs.

#### 5.2.2 Intracellular citrate as a fuel in late stage of bone formation

As BMSC activation occurs, intracellular glucose metabolism generates citrate which eventually produces ATP - becoming the main energy supply pathway. Intracellular citrate production is reliant on the activity of citrate synthestase (CS) (73). Research findings suggest that rats with postmenopausal osteoporosis exhibit a decrease in CS activity (74). In our previous study, we observed a reduction in citrate content in osteoporotic mice through energy metabolism sequencing analysis (75). Moreover, the heightened production of citrate is associated with diminished aconitase (ACO2) activity, which facilitates the conversion of citrate to isocitrate. ACO2 has not been extensively studied as a rate-limiting enzyme in the TCA cycle. However, its function is known to be inhibited by Zn^2+^, and zinc finger protein 1 (ZIP1) aids in Zn^2+^ transportation (48, 76). Furthermore, knockdown of ZIP1 resulted in the prevention of intracellular citrate accumulation, demonstrating that ZIP1 could increase the intracellular levels of citrate (50). Additionally, research on ZIP1 has also revealed that the expression of ZIP1 and intracellular zinc levels increase during the late stages of osteoblastic differentiation, as osteogenic precursors into bone-forming cells (50). Hence, increased levels of intracellular citrate, rather than that extracellular citrate, is required for the late stage of bone formation.

Meanwhile, intracellular citrates is also essential for the normal function of mitochondria. With assistance from mitochondrial complex II (CII), also known as succinate dehydrogenase-ubiquinone oxidoreductase, ATP is generated to support the osteogenic process. Research has identified mutations in succinate dehydrogenase complex A (SDHA) within CII present in osteoblasts from individuals with age-related osteoporosis,SDHA which is nuclear-encoded is a subunit of CII and it is the marker of CII (77), underscoring the significance of energy produced by mitochondria. Abnormal mitochondrial function can adversely affect ATP synthesis and consequently impact osteogenesis. Furthermore, an *in vitro* study demonstrated that induction of osteoblast differentiation progressively enhances the activity of mitochondrial complexes I and II (78). In addition, the absence of mitochondrial citrate may impair the energy supply within the Krebs cycle, which could lead to a failure to meet the bioenergetic energy demands of proliferating and differentiated cells, thereby inhibiting their proliferation and differentiation (48). This suggests that citrate also plays an essential role as a fuel in mitochondria and thereby participates in the osteogenic differentiation of BMSCs.

#### 5.2.3 Citrate as a fuel in bone absorption

Moreover, citrate also holds significance in providing energy for osteoclasts. Mature osteoclasts, rich in mitochondrial DNA, transfer ATP from mitochondria to the cytoplasm. A subsequent increase in ATP levels results in enhanced bone resorption (79). Research has also indicated that during RANKL-stimulated osteoclast differentiation, there is upregulation of CS and other metabolic enzymes to increase citrate synthesis, which is associated with increased production of ATP (80). In another study, it was also demonstrated that the addition of 1-2mM sodium citrate significantly enhances osteoclastogenesis, highlighting the essential role of citrate during osteoclastic differentiation (81). However, there have been relatively few studies on how citrate is involved in the osteoclastic differentiation. More studies are needed to fully understand how energy is utilized during the process of bone resorption.

## 6 Citrate regulates bone remodeling through histone acetylation modification

Citrate not only serves as an energy source but also regulates bone remodeling through the modulation of histone acetylation. Histone protein acetylation refers to the addition of an acetyl group (CH3CO-) to specific amino acid residues, typically lysine, within a histone molecule. This modification process is tightly controlled by enzymes called acetyltransferases and deacetylases. Acetyltransferases transfer acetyl-CoA to amino acid residues, playing a crucial role in modulating histone protein function. The production of acetyl-CoA occurs in mitochondria and its transport to the cytosol heavily relies on the citrate-malate-pyruvate shuttle (82). Mitochondrial citrate carrier (CIC) facilitates the exchange of citrate from mitochondria to cytoplasm with malate. Once in the cytoplasm, citrate is converted into acetyl-CoA by ACLY enzyme, thereby participating in the process of histone acetylation. Research has shown that additional application of citrate promotes higher levels of acetylation (83). Hydroxy-citrate (HCA), which competitively inhibits citrate, hinders this process (84). Acetyl-CoA modulates protein activity through acetylation, particularly influencing histones involved in transcription maintenance during G1 phase and estrogen receptor proteins (ER) responsible for regulating protein homeostasis (85). Unlike nonhistone proteins, histones undergo lysine residue modification at their N-terminal tails through acetylation, causing them to protrude from nucleosomes. Consequently, negatively charged DNA is repelled leading to chromatin relaxation. This open chromatin conformation facilitates easier binding of transcription factors and subsequently influences gene expression (86, 87) (**Figure 2**).

### 6.1 Citrate promotes osteogenic differentiation of BMSC through histone acetylation

Runx2, Sp7, and FoxO1 are crucial transcription factors essential for osteoblast differentiation and maturation. They stimulate the expression of key genes such as ALP, osteocalcin, osteopontin, and COL-1, thereby promoting the maturation and mineralization of osteoblasts. Research indicates that histone H3 acetylation plays a facilitative role in osteoblast differentiation and maturation by enhancing Runx2 transcription (88, 89). Decreased histone acetylation caused by HDAC leads to the suppression of Runx2, SP7, and FoxO1 transcription, ultimately inhibiting osteoblast differentiation and maturation. This suggests that citrate may enhance osteogenic differentiation through modulation of histone acetylation (90–93). In the aging process, mitochondrial structural abnormalities result in reduced CIC levels. Impaired transport of citrate from mitochondria to cytoplasm along with decreased histone acetylation contributes to senile osteoporosis. Exogenous supplementation of acetyl-CoA has been shown to effectively treat osteoporosis; thus, highlighting the importance of citrate decomposition into acetyl-CoA as a critical pathway for regulating acetylation modification during osteogenic differentiation (15).

### 6.2 Citrate promotes osteoclast differentiation through histone acetylation

Citrate regulates histone acetylation to modulate osteoclasts, serving as a crucial mediator of bone resorption. NFATc1 and NF-κB govern osteoclast differentiation (94). Histone H3 acetylation by CBP/p300 enhances the expression of NFATc1 and NF-κB, thereby promoting osteoclast differentiation; conversely, HDAC-mediated H3 deacetylation inhibits this process (88, 89). Furthermore, inhibition of ACLY suppresses osteoclast differentiation and function through its influence on histone acetylation, suggesting that citrate has the potential to enhance osteoclastogenesis via modulation of histone acetylation (95). Osteoclasts’ ability to resorb bone is influenced by their production of reactive oxygen species (ROS). ROS plays a beneficial role in facilitating osteoclast differentiation (96). The scavenging of ROS relies on the action of antioxidant enzymes. One study demonstrated that histone acetylation modification may promote the expression of superoxide dismutase (SOD) (97), while another study showed that histone acetylation can regulate the expression of antioxidant enzyme genes SOD1 and SOD2 (98). Conversely, research suggests that the SOD family contributes to maintaining bone homeostasis by promoting osteoblast differentiation and inhibiting osteoclast differentiation (99–101) This suggests that citrate may promote osteoclast differentiation by modulating histone acetylation, up-regulating transcription factors associated with osteoclastogenesis, and potentially inducing the expression of antioxidants, thereby attenuating oxidative stress and impeding osteoclast differentiation. Overall, citrate significantly contributes to osteoclast formation; however, its inhibition of ROS through histone acetylation may restrict further enhancement of bone resorption. Nevertheless, as mentioned earlier, citrate promotes osteoclast formation *in vitro*, indicating its predominantly promoting effect on bone resorption. In summary, citrate serves as a crucial energy source for ATP production and a substrate for histone acetylation while playing a role in bone remodeling processes mediated by both osteoblasts and osteoclasts. The simultaneous impact on bone formation and resorption poses challenges in assessing its influence on bone mass. Nonetheless, research has demonstrated that exogenous citrate can enhance bone mineral density and mitigate excessive bone resorption in patients (102). Another study demonstrated that exogenous citrate effectively reversed bone resorption in mice (102). As previously mentioned, citrate promotes the proliferation of osteoclasts, indicating its contrasting effects in both *in vivo* and *in vitro* settings. *In vitro*, citrate enhances bone resorption, whereas *in vivo* it suppresses this process. These findings suggest that the primary impact of citrate on bone tissue lies within osteogenesis.

## 7 Citrate regulates disordered bone remodeling through energy reprogramming

### 7.1 Connection between bone remodeling and energy metabolism

The skeletal system is influenced by systemic metabolic processes, including glucose, lipid, and amino acid metabolism. Among the body’s organs, bones rank fourth in terms of glucose consumption, which plays a crucial role in bone development (103). In the presence of oxygen, differentiated cells typically respond to OXPHOS by metabolizing glucose into CO2 and maximizing 5’-adenosine triphosphate (ATP) production (104) to provide energy for cellular activities. Lipid metabolism encompasses the biological processes of fat digestion, absorption, synthesis, and decomposition that are essential for maintaining cellular homeostasis (105). Glutamine metabolism holds particular importance among amino acid metabolisms due to its prevalence in plasma. Apart from facilitating protein biosynthesis directly, glutamine also serves as a vital carbon source and nitrogen donor for nucleotide synthesis, amino acid synthesis, glutathione production, and other essential compound formation (106). Cell metabolism forms the core of the metabolic cycle; thus maintaining a balance between osteoblast and osteoclast metabolism ensures metabolic homeostasis within bone tissue. Any disruption to this balance will result in an imbalance of bone metabolism.

#### 7.1.1 Bone remodeling and glucose metabolism

The regulation of glucose metabolism primarily relies on estrogen and glucose transporters. Estrogen plays a crucial role in maintaining glucose homeostasis (107). Research has demonstrated that osteoporotic rats exhibit decreased systemic glucose metabolism (108). During menopause, estrogen-triggered cellular pathways activate PI3K/AKT-mediated glucose uptake, leading to glucose deprivation (109, 110), which is mediated by the estrogen receptors ESR1 and ESR2 as well as the glucose transporter. Furthermore, the function of transporting glucose into cells cannot be separated from the role of glucose transporters. Glucose is subsequently metabolized in the cytoplasm through glycolysis to produce two molecules of pyruvate, two ATPs, and two NADHs. Among significant glucose transporters facilitating glucose uptake in osteoblast lineage cells are Glut-1, along with Glut-3 and Glut4 (111, 112). Unlike muscle cells, both osteoblasts and osteoclasts take up glucose independently of insulin (113). Glut1 acts as a facilitator for insulin-independent uptake of glucose by transporting it across a concentration gradient. Loss of Glut1 in osteoblast precursors inhibits their differentiation into mature osteoblasts both *in vitro* and *in vivo (* 114). Interestingly, estrogen can also regulate glycometabolism by activating AKT signaling through its receptor ESR1 (115), directly enhancing transcription of the *SLC2A4* gene encoding Glut4 (116), while deficiency in ESR2 reduces AKT expression (117); thus estrogen deficiency may lead to impaired intake disorder mainly due to reduced activation of the AKT pathway mediated by ESR1 and ESR2.

#### 7.1.2 Fatty acid metabolism and bone remodeling

Lipids, including fatty acids, cholesterol, triglycerides (TG), and phospholipids, have increasingly been associated with bone metabolism. Recent research suggests that lipids and their derivatives are significant sources of energy for osteoblasts, shifting the focus from glucose alone. Osteoblasts possess the necessary receptors and catabolic enzymes to uptake and utilize circulating lipids (118). Fatty acids and their derivatives play a crucial role in maintaining bone health, with their levels in the bone microenvironment being linked to osteoporosis (119). Furthermore, fatty acids are known to significantly contribute to osteogenic differentiation as previous studies have demonstrated the ability of osteoblasts to oxidize fatty acids (120). *In vitro* studies have shown a substantial increase in fatty acid oxidation during osteoblast maturation, with mineralized osteoblasts exhibiting three times higher fatty acid catabolic activity compared to proliferating cells This research highlights the crucial role of fatty acids in promoting osteogenic differentiation and mineralization. The uptake and utilization of fatty acids by osteoblasts are primarily facilitated by specific receptors for fatty acid uptake and enzymes involved in fatty acid catabolism. Osteoblasts express the CD36 receptor to facilitate free fatty acid uptake, and studies conducted on mice have demonstrated that depletion of CD36 leads to reduced bone mass due to impaired osteoblast-mediated bone formation (121). Furthermore, osteoblasts possess numerous metabolic enzymes responsible for processing fatty acids. Upon cellular entry, fatty acids are predominantly utilized through fat oxidation and β-oxidation pathways. In the cytoplasm, they are converted into fatty acyl-CoA, which then binds to CPT1—an enzyme located on the outer mitochondrial membrane—to generate acylcarnitine that is subsequently transported into the mitochondrial matrix. Once inside the mitochondria, CPT2 converts acylcarnitine back into acyl-CoA for β-oxidation. Research findings have revealed that knockout of CPT2 results in impaired bone formation (122) Subsequently, acetyl-CoA, NADH, and FADH2 undergo β-oxidation, leading to the generation of fuel for various metabolic pathways. Research findings have demonstrated that pharmacological inhibition of β-oxidation *in vitro* impedes osteoblast differentiation (123). In summary, the transportation and metabolism of fatty acids play a significant role in osteoblasts, while disruptions in fatty acid metabolism in osteoporosis impact the bone formation process of osteoblasts.

#### 7.1.3 Glutamine metabolism and bone remodeling

The regulation of glutamine primarily relies on the cysteine transporter 2 (ASCT2, also known as *SLC1A5*) and aminidase (GLS). ASCT2 facilitates the transport of glutamine from the bloodstream into cells to maintain cellular glutamine homeostasis, while GLS deaminates glutamine to form glutamate, which further undergoes deamination to produce αKG. Studies have indicated that the absence of sodium-dependent amino acid exchanger *SLC1A5* affects the uptake of essential glutamine and asparagine required for maintaining amino acid balance in osteoblasts (124). Research has demonstrated that both glutamine metabolism and GLS activity play a role in mediating osteoblast differentiation (125). Genetic inactivation of GLS1 leads to the elimination of PTH-induced osteoblast generation (126). Previous studies have described age-related changes in glutamine metabolism in osteoporosis, which may disrupt the balance between osteogenic and adipocyte differentiation of BMSC due to impaired key enzymes involved in glutamine metabolism or declining mitochondrial function (127, 128). Recent research has emphasized the potential impact of glutamine metabolism on osteoblast development. Glutamine is crucial for matrix mineralization in osteoblast calvaria cultures. It has been observed that as BMSCs age, their uptake of glutamine significantly decreases, leading to a reduction in osteoblast formation. Isotopic tracing studies have revealed that glutamine is converted into citrate through the TCA cycle, thereby assisting osteoblasts in energy production (129). Furthermore, glutamine exerts an inhibitory effect on osteoclasts, and α- ketoglutarate(α-KG) serves as a metabolite of glutamine. Administration of exogenous α-ketoglutarate has been shown to significantly increase trabecular bone mineral density, cortical bone mineral density, and bone mechanical properties. Moreover, it has been observed to alleviate symptoms associated with osteopenia and osteoporosis in animals and postmenopausal women (130–133). A study discovered that treatment with αKG reduced H3K9me3 levels, resulting in chromatin opening. This subsequently led to decreased RANKL-induced ROS production and inhibition of osteoclast differentiation. These findings indicate the crucial role played by glutamine in regulating osteoclast differentiation (134). In summary, glutamine promotes bone remodeling by stimulating both the proliferation and differentiation of osteoblasts while suppressing the differentiation of osteoclasts through α-KG. Impaired glutamine metabolism due to disorders in bone remodeling can lead to abnormal functioning of both osteoblasts and osteoclasts, thus influencing overall bone remodeling.

### 7.2 Citrate and energy metabolism

**Figure 3:**
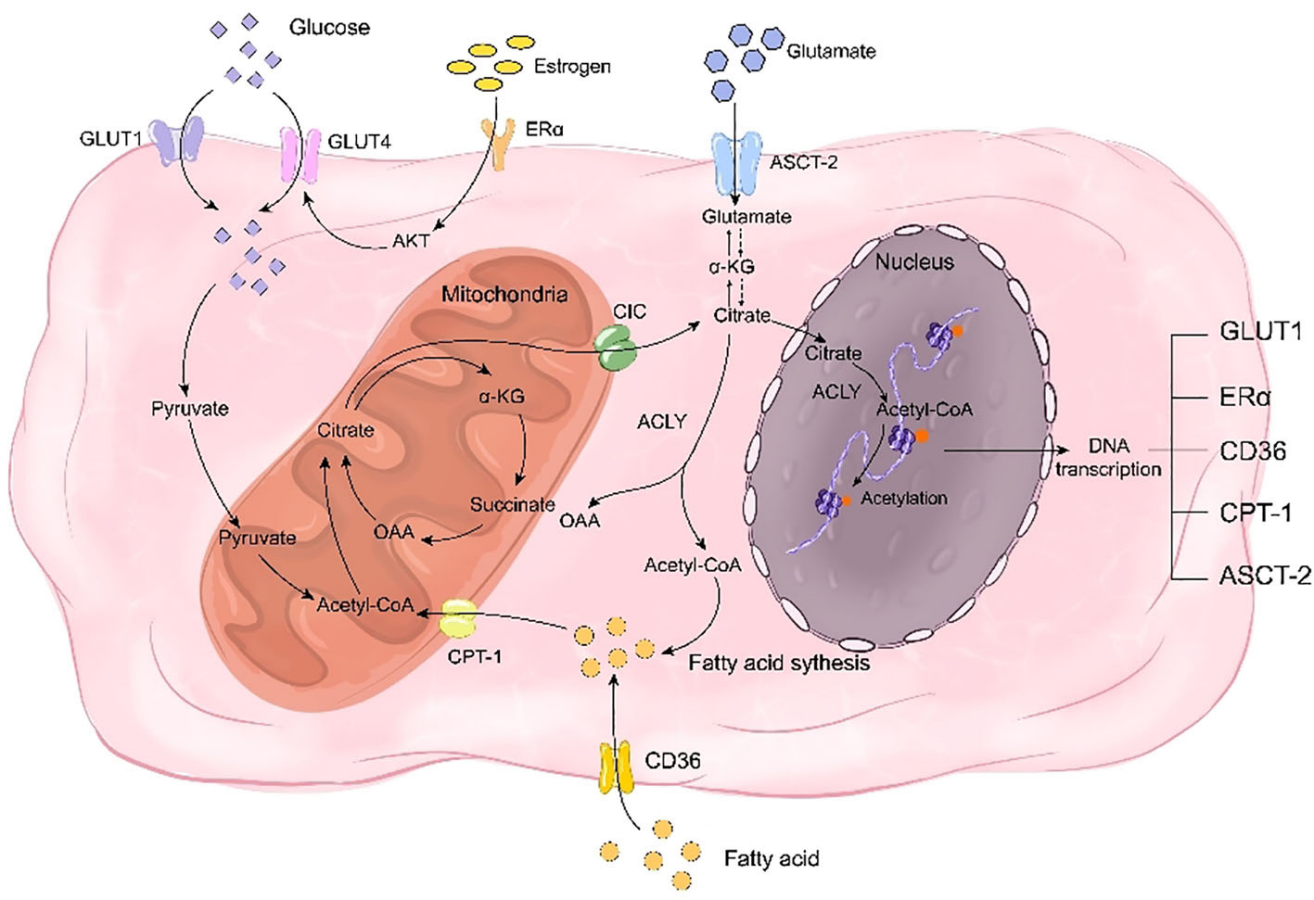
Citrate regulates bone remodeling through energy reprogramming. On one hand, citrate acts as a crucial intermediate in glucose metabolism, while on the other hand, it facilitates the synthesis of fatty acids and glutamine to compensate for deficiencies in glucose, fatty acids, and glutamine during bone remodeling disorders. Citrate promotes the transcription of transporters for these three nutrients, including GLUT1, CD36, and ASCT-2, thereby facilitating their uptake. Additionally, citrate enhances histone acetylation to promote the transcription of estrogen receptor ERα and mitigate the impact of estrogen deficiency on glucose transporter GLUT4. Simultaneously, citrate upregulates CPT-1 transcription through histone acetylation to enhance fatty acid metabolism and play a pivotal role in rescuing energy metabolism disorders.

Ordinarily, the three metabolic types in normal individuals are interconnected. However, osteoporosis-related bone remodeling disorders can disrupt glucose, fatty acid, and glutamine metabolism, resulting in a metabolic disorder known as reprogramming. Reprogramming involves altering cellular or tissue metabolic pathways that lead to changes in cell function and physiological status. Citrate plays a critical role in this process (19). Citrate-mediated metabolic reprogramming can treat bone metabolic disorders by supplying essential intermediates for glucose and fatty acid metabolism. In glucose metabolism, citrate serves as a crucial intermediate in the TCA cycle while contributing to fatty acid synthesis by transporting acetyl-CoA into the cytoplasm through its involvement in the citrate-pyruvate cycle (135). In the context of glutathione metabolism, citrate can participate in the synthesis of glutamine despite being a crucial intermediate in glutamine metabolism (136). Furthermore, citrate also exerts regulatory control over metabolic reprogramming by modulating estrogen receptors and key metabolic proteins (**Figure 3**).

#### 7.2.1 Citrate regulates glucose metabolism to mediate bone remodeling

Exogenous citrate has the potential to ameliorate glucose metabolic disorders. As an intermediate in the TCA cycle, citrate can partially compensate for energy metabolic disorders caused by glucose deficiency. Moreover, it enhances glucose homeostasis by augmenting glucose uptake. A study demonstrated that exogenous chromium citrate significantly increased glucose intake in rats and upregulated Glut4 expression and AMPK transcription, indicating that citrate could enhance cellular glucose uptake and improve bone homeostasis through increased expression of glucose transporters (137). Another study revealed that HDAC inhibitors promote GLUT1 acetylation to facilitate glucose uptake, suggesting another mechanism by which citrate directly promotes glucose uptake via acetylation to enhance transporter expression (138). Additionally, a separate study illustrated that estrogen receptor ESR undergoes acetylation and histone H3 lysine 27 (H3K27ac) acetylation can regulate promoter regions of ER pathway genes encoding ERα and ESR1 (139). Therefore, the upregulation of ESR estrogen receptor expression induced by exogenous citrate may also serve as a possible mechanism through which it improves glucose uptake in osteoporotic bone tissue.

#### 7.2.2 Citrate-mediated fatty acid metabolism regulates bone remodeling

Citrate is a crucial substrate for fatty acid synthesis and serves as the initial component, acetyl-CoA, in the pathway. After being transported out of the cell by CIC, citrate undergoes breakdown into acetyl-CoA. Subsequently, through successive reactions involving enzymes such as acetyl-CoA carboxylase, dehydratase, reductase, and acylase (140), it can further synthesize long-chain fatty acids. CICs have been proven to promote fatty acid biosynthesis (141). Additionally, the expression of SLC25A1, a type of mitochondrial citrate transporter, was found to be most prominent in adipogenic tissues such as the liver, renal cortex, and pancreas (142). As fatty acids are essential substrates for adipogenesis and CIC transports citrate accordingly plays a significant role in fatty acid production. Furthermore, citrate has the ability to influence histone acetylation and thereby impact fatty acid transport and metabolism. A proteomic examination of lysine acetylation sites in mouse and rat tissues identified CD36 as being acetylated at lysine 52, 166, 231, and 403 (143). Mass spectrometry analysis confirmed the acetylation of these sites on human CD36 (144). This finding suggests that histone acetylation-mediated upregulation of CD36 transcription may contribute to the elevated intracellular fatty acid levels. Additionally, another study demonstrated that enhanced acetylation of mitochondrial fatty acid β-oxidase promotes fatty acid breakdown (145). Moreover, it was observed that CPT-1, an enzyme responsible for transporting fatty acids into mitochondria, is susceptible to acetylation. These findings collectively indicate that citrate not only serves as a precursor for fatty acid synthesis but also plays a crucial role in regulating fatty acid uptake, breakdown, and β-oxidation to support osteoblast differentiation and mineralization. In summary, citrate acts as a modulator for proteins involved in fatty acid transport and metabolism in osteoblasts, facilitating processes such as absorption, synthesis, breakdown, and oxidation of fatty acids while maintaining their homeostasis.

#### 7.2.3 Citrate-mediated glutamine metabolism regulates bone remodeling

Citrate can enhance the regulation of amino acid metabolism disorder by modulating glutamine synthesis and transport. CIC deficiency has been shown to impair glutamine synthesis, indicating a positive correlation between cytoplasmic citrate content and glutamine levels (142, 146). Additionally, *SLC25A1* mutant mice with hydroxyglutaric aciduria exhibited altered glutamine remodeling (147). In Huh7 cells deprived of glutamine, supplementation with exogenous citrate rescued cell viability that was reduced by NaCT inhibition, suggesting that exogenous citrate could restore depleted glutamine levels via NaCT (148). Furthermore, studies have demonstrated that exogenous citrate generates derivatives of both glutamine and glutamate as well as promotes fatty acid synthesis (138), implying its potential in regulating the content of glutamine to ameliorate amino acid metabolism disorders in osteoporosis patients. Moreover, citrate can modulate ASCT2-mediated uptake of glutamine; previous research indicated that bortezomib (BTZ)-induced peripheral neuropathy led to decreased histone acetylation which may silence *SLC1A5* expression (149), while another study suggested upregulation of ASCT2 in mice subjected to chronic social defeat stress due to excessive histone acetylation (150). These findings suggest that citrate can promote ASCT2 expression and enhance the uptake of glutamine. Therefore, citrate plays a regulatory role in bone remodeling by increasing the content of intracellularly available glutamine and modulating its intake as well as metabolism.

## 8 Citrate regulates bone remodeling through the immune system

The link between bones and the immune system holds significant importance. The immune system comprises immune organs, immune cells, and immune factors, while bone homeostasis involves osteoblasts and osteoclasts. Extensive descriptions have been provided regarding the role of immune inflammation in bone loss. In cases of pathological immune dysfunction, such as immune deficiency or inflammatory response to infection/disease, the bone is impacted by the immune response, potentially leading to osteoporosis and an increased risk of fracture (151, 152). Moreover, various prevalent inflammatory conditions exacerbate bone loss including rheumatoid arthritis, periodontal infection, and inflammatory bowel disease. Immune-inflammatory factors are considered as the primary means through which the immune system regulates bone remodeling. Postmenopausal women with osteoporosis often exhibit a chronic mild inflammatory state characterized by altered cytokine expression (153). Citrate plays a crucial role in modulating the release of inflammatory factors; thus suggesting that citrate’s impact on these factors may serve as a mechanism for regulating bone remodeling.

### 8.1 Bone immune factors and bone remodeling

The immunophenotyping clinical evidence in postmenopausal patients indicates that women in the postmenopausal stage exhibit elevated levels of inflammatory cytokines, specifically interleukin-1β (IL-1β), IL-6, and tumor necrosis factor α (TNFα) (154–157). This applies to both circulating blood cells and cells within the bone microenvironment (158, 159). Previous experimental evidence confirms the heightened presence of inflammatory mediators, such as IL-1β, IL-6, and TNFα, in the bloodstream and bone marrow of ovariectomized rodents (160, 161). In summary, postmenopausal women with osteoporosis exhibit a persistent mild inflammatory state characterized by altered cytokine expression. This suggests that estrogen-mediated immune cell-induced changes in inflammatory cytokines play a crucial role in bone formation.

The regulation of bone immunity primarily involves three inflammatory factors, namely IL-1β, IL-6, and TNF-α. IL-1β, a prominent member of the IL-1 ligand family, is recognized as a primary therapeutic target for various inflammatory conditions (162). Multiple studies have demonstrated that IL-1 directly enhances osteoclast formation, multinucleation, pit-forming activity, and survival (163). Additionally, IL-1 also affects osteoblasts. In a study by Zhang YZ et al., downregulation of TLR4 reduced the levels of IL-1, TNF-α, and IL-6 in osteoblasts leading to improved cell viability attributed to the suppression of inflammatory pathways (164). Furthermore, IL-1β stimulates bone resorption and inhibits bone formation while IL-6 promotes T cell growth and differentiation and enhances the differentiation of osteoclasts, macrophages, and megakaryocytes (165). Moreover, IL‐6 has dual effects on osteoblast activity by promoting initial differentiation but impeding subsequent differentiation at later stages (166–168) TNF-α, an inflammatory factor, can be produced by various cell types such as macrophages, NK cells, mast cells, and T and B lymphocytes (169). The impact of TNF-α on osteogenic differentiation remains a subject of debate. Some studies suggest that lower concentrations of TNFα enhance the levels of Runx2, Osx, OCN, and ALP in MSCs (170, 171), while higher concentrations of TNFα have been shown to decrease these levels (170–173). In terms of the relationship between TNF-α and osteoclasts, it is considered a crucial stimulator for osteoclast differentiation (174). Treatment with TNFα alone (without RANKL) has been found to increase the number of TRAP-positive osteoclasts in WT mice by activating the NF-κB signaling pathway both locally and systemically (175–177). Overall, inflammatory factors predominantly regulate bone remodeling by inhibiting osteogenic differentiation and promoting osteoclast differentiation.

### 8.2 Citrate regulates immune inflammatory factors to regulate bone remodeling

**Figure 4:**
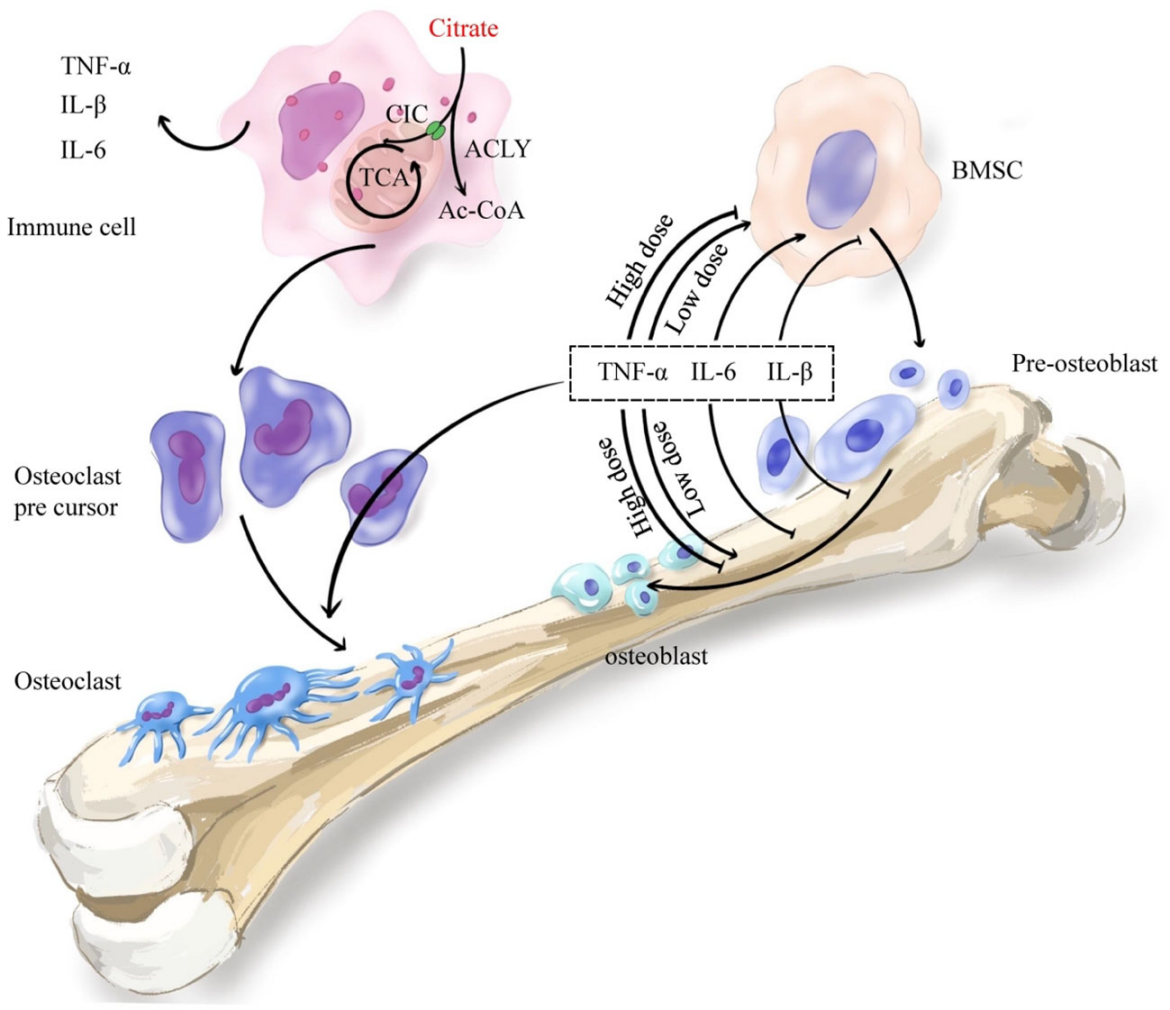
Citrate regulates bone remodeling through the immune system. Citrate regulates histone acetylation in immune cells through ACLY and promotes the secretion of immune inflammatory factors TNF-α, IL-1β and IL-6. The secreted immune inflammatory factors promote the differentiation of osteoclast precursor cells into osteoclasts which mediate bone resorption. Meanwhile, the inflammatory cytokines TNF-α, IL-1β and IL-6 also play an important role in bone formation. TNF-α promoted osteogenic differentiation at low concentrations and inhibited osteogenic differentiation at high concentrations. IL-6 promoted early differentiation and inhibited late differentiation. IL-1β mainly inhibited osteogenic differentiation. In general, increased immune-inflammatory factors enhance bone resorption and inhibit bone formation.

Citrate plays a pivotal role in modulating immune factors. Numerous studies have demonstrated that exogenous citrate influences the secretion of IL-1, TNF-α, and IL-6 (**Figure 4**). A study indicated that the introduction of exogenous citrate resulted in elevated expression of proinflammatory cytokines TNF-α, IL-1β, and IL-6 (178). Research conducted on gastric cancer epithelial cells demonstrated that citrate could enhance the expression of IL-1β and TNF-α (179). Furthermore, another study revealed a positive correlation between increased plasma levels of ACLY and the expression of IL-6, suggesting that citrate may promote the expression of immune factors through ACLY. ACLY breaks down citrate into acetyl-CoA, which serves as a substrate for acetylation - a fundamental mechanism for regulating immune factors. In one study, it was demonstrated that the absence of ACLY led to reduced secretion of IL-6 and TNF-α in macrophages primarily due to decreased macrophage response to IL-4 stimulation caused by reduced levels of histone acetylation-dependent genes targeted by IL-4 (180, 181). Another study also demonstrated that silencing of SLC25A1 suppressed the production of TNF-α induced by LPS (182). The results indicated a positive correlation between cytoplasmic citrate levels and the expression of IL-1β, TNF-α, and IL-6. Ethanol-exposed monocyte-macrophages convert it to acetate, which metabolizes into acetyl-CoA, leading to enhanced histone acetylation and generation of proinflammatory cytokines such as IL-6, IL-8, and TNF-α. These cytokines were influenced by the downregulation of ACSS1 and ACSS2 from the short-chain family members of acyl-CoA synthetase (183, 184). These findings suggested that ACLY activity resulted in the synthesis of acetyl-CoA from citrate, thereby facilitating the expression of inflammatory factors while regulating bone formation and resorption. In brief,the inhibition of bone formation is facilitated by citrate through the upregulation of inflammatory factor expression.

## 9 Citrate as a basis for the diagnosis and evaluation of therapeutic effect of bone remodeling disorder

Due to its significant impact on bone remodeling and resorption, citrate emerges as a potential molecular marker for various orthopedic-related disorders. Research has revealed a marked reduction in citrate levels in the bones of mice or rats experiencing bone loss induced by age-related ovariectomy or retinoic acid, indicating an association between citrate and the development and progression of diverse forms of osteoporosis. Our previous energy metabolomics study also found that the citrate content of bone tissue was weakened in postmenopausal osteoporotic mice (23, 185). Currently, due to limited approaches for acquiring bone tissue samples, research on bone tissue citrate is primarily restricted to animal experiments, while studies on human citrate and osteoporosis mainly focus on plasma and urine citrate levels. A study revealed notable decreases in plasma citrate levels among elderly osteoporotic men compared to younger healthy men, suggesting that reduced citrate levels in osteoporotic patients may not solely be attributed to estrogen (23). Simultaneously, research has demonstrated a positive correlation between plasma citrate and lumbar hip BMD (23). The findings suggest that serum citrate levels have the potential to serve as a diagnostic marker for osteoporosis. Although urine testing offers the advantages of noninvasiveness and convenience compared to blood testing, a study has demonstrated decreased citrate excretion in postmenopausal women and individuals with low bone mass (186, 187). Additionally, there is strong evidence linking urinary citrate excretion to prevalence of fragility fractures in postmenopausal women (28), indicating a potential association between urinary citrate levels and bone mineral density. Importantly, the administration of potassium citrate to increase urinary citrate excretion, typically used for stone prevention purposes, resulted in a slight improvement in lumbar bone mineral density - an intriguing finding (188). This suggests that urine citrate could also be considered as a potential diagnostic marker for osteoporosis. However, further research is needed to determine whether plasma or urine citrate is superior and if combining both blood and urine measurements can enhance the accuracy of diagnosing osteoporosis.

Citrate may also serve as a biomarker for assessing the therapeutic effect of osteoporosis. Numerous drug studies have demonstrated that promoting citrate secretion can effectively treat osteoporosis. Melatonin plays a pivotal role in the management of osteoporosis, as evidenced by its ability to enhance citrate secretion and increase citrate content in bone tissue when treating mouse osteoblasts. This suggests that citrate could potentially serve as an indicator for evaluating the prognosis of melatonin-treated osteoporosis (189). Traditional Chinese medicine (TCM) is also an important treatment modality for osteoporosis. One study suggested that osthole regulates blood citrate concentration to treat osteoporosis (185). Another study indicated that the citrate cycle might be a crucial mechanism through which Chinese medicine (Yigu decoction) regulates intestinal flora to address the pathogenesis of osteoporosis, implying that citrate could be a vital prognostic marker for its treatment (190). Previous studies conducted by our group have found evidence supporting melatonin’s ability to regulate intestinal flora and promote citrate secretion, suggesting its potential role in treating osteoporosis. In conclusion, while further confirmation is needed regarding its accuracy, citrate holds promise as an indicator for evaluating the efficacy of medical treatments targeting osteoporosis.

## 10 Citrate-based treatment of bone remodeling disorders

### 10.1 Citrate diet corrects bone remodeling and bone metabolism disorders

Since citrate production in the human body primarily relies on dietary intake, consuming foods rich in citrate is the preferred approach for correcting bone remodeling. Research suggests that incorporating citrate-rich fruits and vegetables into one’s diet promotes renal excretion of citrate (191). Interestingly, compared to individuals with normal fruit intake, those with lower fruit consumption exhibit reduced levels of endogenous citrate content (192), indicating that exogenous sources of citrate compensate for decreased renal excretion and play a role in correcting bone remodeling. A study demonstrated that potassium citrate significantly increased bone mass in postmenopausal women (193). Another study revealed that supplementation with potassium citrate reduced markers of bone resorption (194). However, a two-year clinical trial indicated that simply increasing oral citrate dosage did not improve bone remodeling (195). This inconsistency may arise from the fact that osteoblast secretion primarily contributes to the presence of citrate in bones (23). External sources of citrate are absorbed into the bloodstream and eliminated by the kidneys, thereby maintaining serum citrate homeostasis without affecting its levels within bone tissue. Consequently, strategies aimed at enhancing bone formation through elevated levels of intracellular or extracellular citrates have become a focal point.

### 10.2 The important role of citrate scaffold in the treatment of bone loss

Currently, orthopedic biomaterials developed for bone regeneration lack the necessary biological and biochemical compatibility required for effective and intricate bone healing. However, bone regeneration plays a crucial role in clinical care for various conditions such as nonunion defects, congenital anomalies, traumatic injuries, and tumor removal (196). Citrate-based scaffolds have been extensively utilized as orthopedic materials. Numerous studies have demonstrated that the incorporation of citrate into biomaterials enhances bone formation (197–199). Simultaneously, with the advancement of research on bone regeneration materials, researchers generally aim to provide not only physical filling but also biological support for bone tissue regeneration through scaffolds. Previous research has shown that citrate-based materials serve as an energy source for human stem cells and stimulate their osteogenic differentiation via metabolic control, which is dependent on NaCT (69). The efficacy of heat-responsive citrate-based GO scaffolds in promoting BMP9-stimulated bone regeneration in adipose-derived MSCs has been demonstrated by another study (200). These findings suggest that citrate scaffolds not only enhance osteogenesis by promoting the differentiation of stem cells into osteoblasts but also expedite mineralization through facilitating hydroxyapatite deposition (201). Moreover, research has indicated that citrate biomaterials possess inherent antibacterial properties, which can inhibit bone regeneration during infection post bone defects. Many biodegradable polymers based on citrate have shown notable antibacterial activity against Gram-negative Escherichia coli and Gram-positive Staphylococcus aureus (202, 203). Additionally, blood circulation plays a crucial role in the healing process of bone defects, and it has been found that citrate materials can improve blood compatibility (204) Interestingly, the angiogenic impact of citrate varies depending on the dosage. Research suggests that lower doses of citrate material promote osteogenic differentiation while higher doses further stimulate angiogenesis in vascular endothelial cells (205). Furthermore, citrate provides an additional binding site for biological conjugation (206) improving scaffold biocompatibility (201) as well as enhancing hydrophilicity and cell adhesion. In conclusion, through multiple biological pathways (207), citrate enhances the functionality of filled scaffolds in bone formation.

### 10.3 Targeting citrate transporters may be a new direction for drug development of bone remodeling in the future

For patients with bone defects, citrate scaffolds have demonstrated satisfactory efficacy in promoting bone regeneration as fillings. However, the utilization of citrate scaffolds is not applicable for non-traumatic metabolic bone diseases such as osteoporosis. Notably, the angiogenic impact of citrate has been found to be dose-dependent. Research has shown that at low doses, citrate materials induce osteogenic differentiation while at high doses they enhance the angiogenic function of vascular endothelial cells. As mentioned earlier, NaCT and CIC serve as transporters for citrate, with NaCT responsible for cellular absorption and CIC facilitating transportation from mitochondria to the nucleus. Consequently, exploring potential medications targeting citrate transporters presents a promising approach to address disorders related to bone remodeling by regulating citrate levels in bone tissue. The current drugs targeting citrate transporters primarily consist of transporter inhibitors. PF-06649298 inhibits NaCT (208). By binding to NaCT and inhibiting citrate uptake in human hepatocytes, PF-06649298 reduced plasma glucose levels and hepatic TG in mice fed a high-fat diet (209). Slc25a1-specific inhibitors (CTPI-2) effectively hinders the progression of nonalcoholic steatohepatitis by reversing significant changes in steatosis, preventing its development into steatohepatitis, reducing infiltration of inflammatory macrophages in liver and adipose tissue, and notably mitigating obesity induced by a high-fat diet (210, 211) Furthermore, studies have demonstrated that inhibitors targeting the citrate transporter CIC exacerbate bone loss (15). Targeting citrate transporters holds promise for the treatment of osteoporosis. Moreover, research indicates that Paget’s Disease of Bone involves abnormal proliferation of bone tissue, leading to fragility and susceptibility to pain and fractures. Elevated plasma citrate levels in patients with Paget’s Disease are associated with increased citrate production (212). This suggests a potential association between Paget’s Disease and excessive citrate overproduction, indicating that inhibitors targeting citrate transporters may be a viable option for drug treatment. Additionally, it has been reported that elderly osteoporosis patients exhibit decreased expression of CIC and NaCT. Therefore, developing agonists for CIC and NaCT in osteoporosis patients could potentially regulate bone resorption and remodeling disorders. Notably, NaCT can enhance the uptake of citrate by osteoblasts. A study has also revealed that reduced expression of CIC leads to mitochondrial dysfunction in mice with senile osteoporosis (15) Therefore, the development of CIC agonists can not only enhance cytosolic citrate content but also ameliorate mitochondrial dysfunction and promote metabolic reprogramming of osteoblasts. Consequently, CIC agonists offer greater advantages over NaCT (17, 211).

## 11 Discussion

In this review, we suggest that citrate plays a critical role in bone remodeling, serving as both a vital by product of the TCA cycle and an indispensable factor in bone mineralization. The role of this substance extends beyond its function as a fuel for bone formation and resorption. It also plays a systemic regulatory role in bone remodeling through mechanisms such as histone acetylation, metabolic reprogramming, and the modulation of pro-inflammatory factors involved in bone immunity. Under physiological conditions, citrate plays a crucial role in bone remodeling by facilitating ATP production and ensuring active transcription through histone acetylation. It also regulates glucose metabolism, fatty acid metabolism, and glutamine utilization to support the synthesis of various necessary substances via metabolic reprogramming. Additionally, it maintains a stable mechanism for bone formation and resorption while inhibiting excessive bone remodeling through up-regulation of immune factors. Under the pathological condition of osteoporosis, the reduction of citrate in bone tissue leads to impaired bone mineralization, disrupted energy metabolism, and decreased histone acetylation.

Interestingly, citrate plays a paradoxical role in bone remodeling. As mentioned earlier, citrate levels significantly impact the process of bone mineralization, with both deficiencies and excesses having detrimental effects on the formation of bones,which suggests that citrate plays a positive role in maintaining bone remodeling.Exogenous citrate has been found to effectively enhance bone density while inhibiting bone resorption. However, citrate can also stimulate the release of immuno-inflammatory factors, which impact bone health[. Recent studies suggest that the immune system’s regulation of bone metabolism is highly complex, and osteoimmunology is an emerging discipline that explores the influence of the immune system on bone tissue (213). This article proposes that circulating citrate may promote the release of inflammatory factors through mechanisms such as histone acetylation, indicating that citrate modulates the overall immune level, which could be detrimental to bone tissue. This contradiction might be explained by the differential functions of citrate within bone tissue at different locations in the body: citrate within bone tissues may help maintain bone remodeling, while circulating citrate may upregulate systemic inflammation levels by modulating the immune system. It seems that citrate within bone tissues plays a more significant role than circulating citrate, as high serum concentrations of citrate are often associated with excessive osteogenesis rather than bone resorption caused by circulating citrate-induced inflammatory factors. Therefore, future research should focus on the differing roles of citric acid at various locations. This will contribute to a deeper understanding of the paradoxical role of citric acid in bone remodeling.

Currently, the assessment of citrate content in the human body primarily focuses on serum citrate levels. Blood citrate level may serve as an indicator of bone metabolic status. This reliance on blood analysis stems from the challenges associated with obtaining bone tissue samples for diagnosing osteoporosis. Additionally, renal processes play a significant role in maintaining citrate homeostasis as mentioned earlier, while urinary excretion of citrate decreases in postmenopausal osteoporosis patients (186). Therefore, urine citrate can also serve as an indicator for assessing bone metabolism in humans (214). While citrate is crucial for maintaining bone health, supplementing with citrate alone may not yield significant effects due to its renal metabolism. Recent studies propose that targeting enzymes associated with citrate metabolism could be a potential therapeutic approach for treating osteoporosis by increasing the content of citrate in bones (15, 45). However, it is important to note that citrate plays a pivotal role in tumor metabolism (15), promoting tumor stemness and drug resistance (211). Citrate assumes a prominent role as a promoter of tumor growth, particularly in intraosseous metastatic tumors where its influence is pronounced (215). Meanwhile, circulating citrates might have negative effects on bone health as is mentioned above. Hence, drugs targeting enzymes related to citrate must specifically target BMSC or osteoblast cells to effectively treat osteoporosis and avoiding any negative impact on bone and also other tissues.

In conclusion, this research emphasizes the significance of citrate in bone metabolism, illustrates the systemic regulation of citrate in bone remodeling, and unveils its multifaceted role in mineralization, energy provision, histone acetylation, and immune response. Furthermore, it is suggested that citrate maintains homeostasis during physiological conditions and prevents excessive bone formation while intrinsic mechanisms prevent further exacerbation of pathological bone resorption. The determination of blood and urine citrate levels under different physiological and pathological conditions may offer a novel method for diagnosing osteoporosis and evaluating treatment efficacy. Additionally, we demonstrate that targeting the citrate transporter presents a promising approach for managing bone remodeling disorders and introduces innovative concepts for orthopedic diagnosis and management.

## References

[1] Yang J Andre P Ye L Yang YZ. The Hedgehog signalling pathway in bone formation. Int J Oral Sci. (2015) 7:73–9. doi: 10.1038/ijos.2015.14 PMC4817553 26023726

[2] Xie H Sun M Liao XB Yuan LQ Sheng ZF Meng JC. Estrogen receptor α36 mediates a bone-sparing effect of 17β-estrodiol in postmenopausal women. J Bone Mineral Res. (2011) 26:156–68. doi: 10.1002/jbmr.169 PMC3179309 20578216

[3] Yewle JN Puleo DA Bachas LG. Enhanced affinity bifunctional bisphosphonates for targeted delivery of therapeutic agents to bone. Bioconjugate Chem. (2011) 22:2496–506. doi: 10.1021/bc2003132 PMC3247145 22073906

[4] Xing Z Lu C Hu D Yu YY Wang X Colnot C. Multiple roles for CCR2 during fracture healing. Dis Models Mech. (2010) 3:451–8. doi: 10.1242/dmm.003186 PMC2898536 20354109

[5] Goltzman D. Discoveries, drugs and skeletal disorders. Nat Rev Drug Discovery. (2002) 1:784–96. doi: 10.1038/nrd916 12360256

[6] Hayden RS Fortin JP Harwood B Subramanian B Quinn KP Georgakoudi I. Cell-tethered ligands modulate bone remodeling by osteoblasts and osteoclasts. Adv Funct Mater. (2014) 24:472–9. doi: 10.1002/adfm.201302210 PMC4235974 25419210

[7] Dirckx N Moorer MC Clemens TL Riddle RC. The role of osteoblasts in energy homeostasis. Nat Rev Endocrinol. (2019) 15:651–65. doi: 10.1038/s41574-019-0246-y PMC6958555 31462768

[8] Da W Tao L Zhu Y. The role of osteoclast energy metabolism in the occurrence and development of osteoporosis. Front Endocrinol. (2021) 12:675385. doi: 10.3389/fendo.2021.675385 PMC8150001 34054735

[9] Dobson PF Dennis EP Hipps D Reeve A Laude A Bradshaw C. Mitochondrial dysfunction impairs osteogenesis, increases osteoclast activity, and accelerates age related bone loss. Sci Rep. (2020) 10:11643. doi: 10.1038/s41598-020-68566-2 32669663 PMC7363892

[10] Tao J Miao R Liu G Qiu X Yang B Tan X. Spatiotemporal correlation between HIF-1α and bone regeneration. FASEB J. (2022) 36:e22520. doi: 10.1096/fj.202200329RR 36065633

[11] Taubmann J Krishnacoumar B Böhm C Faas M Müller DIH Adam S. Metabolic reprogramming of osteoclasts represents a therapeutic target during the treatment of osteoporosis. Sci Rep. (2020) 10:21020. doi: 10.1038/s41598-020-77892-4 33273570 PMC7713370

[12] Lerner UH:. Inflammation-induced bone remodeling in periodontal disease and the influence of post-menopausal osteoporosis. J Dental Res. (2006) 85:596–607. doi: 10.1177/154405910608500704 16798858

[13] Sharma G Sultana A Abdullah KM Pothuraju R Nasser MW Batra SK. Epigenetic regulation of bone remodeling and bone metastasis. Semin Cell Dev Biol. (2024) 154:275–85. doi: 10.1016/j.semcdb.2022.11.002 PMC10175516 36379849

[14] Wang R Wang Y Zhu L Liu Y Li W. Epigenetic regulation in mesenchymal stem cell aging and differentiation and osteoporosis. Stem Cells Int. (2020) 2020:8836258. doi: 10.1155/2020/8836258 32963550 PMC7501554

[15] Pouikli A Parekh S Maleszewska M Nikopoulou C Baghdadi M Tripodi I. Chromatin remodeling due to degradation of citrate carrier impairs osteogenesis of aged mesenchymal stem cells. Nat Aging. (2021) 1:810–25. doi: 10.1038/s43587-021-00105-8 PMC10154229 37117628

[16] Iacobazzi V Infantino V. Citrate–new functions for an old metabolite. Biol Chem. (2014) 395:387–99. doi: 10.1515/hsz-2013-0271 24445237

[17] Icard P Simula L Zahn G Alifano M Mycielska ME. The dual role of citrate in cancer. Biochim Biophys Acta Rev Cancer. (2023) 1878:188987. doi: 10.1016/j.bbcan.2023.188987 37717858

[18] Dreier DA Mello DF Meyer JN Martyniuk CJ. Linking mitochondrial dysfunction to organismal and population health in the context of environmental pollutants: progress and considerations for mitochondrial adverse outcome pathways. Environ Toxicol Chem. (2019) 38:1625–34. doi: 10.1002/etc.4453 PMC6961808 31034624

[19] Williams NC O’Neill LAJ. A role for the krebs cycle intermediate citrate in metabolic reprogramming in innate immunity and inflammation. Front Immunol. (2018) 9:141. doi: 10.3389/fimmu.2018.00141 29459863 PMC5807345

[20] Santarsiero A Leccese P Convertini P Padula A Abriola P D’Angelo S. New insights into behçet’s syndrome metabolic reprogramming: citrate pathway dysregulation. Mediators Inflammation. (2018) 2018:1419352. doi: 10.1155/2018/1419352 PMC6046129 30050389

[21] Ohanele C Peoples JN Karlstaedt A Geiger JT Gayle AD Ghazal N. Mitochondrial citrate carrier SLC25A1 is a dosage-dependent regulator of metabolic reprogramming and morphogenesis in the developing heart. bioRxiv: preprint Server Biol. (2023) 7(1):1422. doi: 10.1101/2023.05.22.541833 PMC11528069 39482367

[22] Mosaoa R Kasprzyk-Pawelec A Fernandez HR Avantaggiati ML. The mitochondrial citrate carrier SLC25A1/CIC and the fundamental role of citrate in cancer, inflammation and beyond. Biomolecules. (2021) 11:141. doi: 10.3390/biom11020141 33499062 PMC7912299

[23] Chen H Wang Y Dai H Tian X Cui ZK Chen Z. Bone and plasma citrate is reduced in osteoporosis. Bone. (2018) 114:189–97. doi: 10.1016/j.bone.2018.06.014 29929041

[24] Jehle S Hulter HN Krapf R. Effect of potassium citrate on bone density, microarchitecture, and fracture risk in healthy older adults without osteoporosis: a randomized controlled trial. J Clin Endocrinol Metab. (2013) 98:207–17. doi: 10.1210/jc.2012-3099 23162100

[25] Haleblian GE Leitao VA Pierre SA Robinson MR Albala DM Ribeiro AA. Assessment of citrate concentrations in citrus fruit-based juices and beverages: implications for management of hypocitraturic nephrolithiasis. J Endourol. (2008) 22:1359–66. doi: 10.1089/end.2008.0069 18578663

[26] Penniston KL Nakada SY Holmes RP Assimos DG. Quantitative assessment of citric acid in lemon juice, lime juice, and commercially-available fruit juice products. J Endourol. (2008) 22:567–70. doi: 10.1089/end.2007.0304 PMC2637791 18290732

[27] Schuster E Dunn-Coleman N Frisvad JC Van Dijck PW. On the safety of Aspergillus Niger–a review. Appl Microbiol Biotechnol. (2002) 59:426–35. doi: 10.1007/s00253-002-1032-6 12172605

[28] Caudarella R Vescini F Buffa A Stefoni S. Citrate and mineral metabolism: kidney stones and bone disease. Front Biosci. (2003) 8:s1084–1106. doi: 10.2741/1119 12957820

[29] Zuckerman JM Assimos DG. Hypocitraturia: pathophysiology and medical management. Rev Urol. (2009) 11:134–44. PMC2777061 19918339

[30] Costello LC Franklin RB. Plasma citrate homeostasis: how it is regulated; and its physiological and clinical implications. An important, but neglected, relationship in medicine. HSOA J Hum Endocrinol. (2016) 1:005. doi: 10.24966/HE-9640/100005 28286881 PMC5345696

[31] Sakhaee K Alpern R Poindexter J Pak CY. Citraturic response to oral citric acid load. J Urol. (1992) 147:975–6. doi: 10.1016/S0022-5347(17)37437-2 1552616

[32] Dickens F:. The citric acid content of animal tissues, with reference to its occurrence in bone and tumour. Biochem J. (1941) 35:1011–23. doi: 10.1042/bj0351011 PMC1265599 16747445

[33] Murshed M. Mechanism of bone mineralization. Cold Spring Harbor Perspect Med. (2018) 8:a031229. doi: 10.1101/cshperspect.a031229 PMC6280711 29610149

[34] Clarke B:. Normal bone anatomy and physiology. Clin J Am Soc Nephrol: CJASN. (2008) 3 Suppl 3:S131–139. doi: 10.2215/CJN.04151206 PMC3152283 18988698

[35] Xie B Nancollas GH. How to control the size and morphology of apatite nanocrystals in bone. Proc Natl Acad Sci United States America. (2010) 107:22369–70. doi: 10.1073/pnas.1017493108 PMC3012503 21169505

[36] Wilson EE Awonusi A Morris MD Kohn DH Tecklenburg MM Beck LW. Three structural roles for water in bone observed by solid-state NMR. Biophys J. (2006) 90:3722–31. doi: 10.1529/biophysj.105.070243 PMC1440753 16500963

[37] Hu YY Rawal A Schmidt-Rohr K. Strongly bound citrate stabilizes the apatite nanocrystals in bone. Proc Natl Acad Sci United States America. (2010) 107:22425–9. doi: 10.1073/pnas.1009219107 PMC3012505 21127269

[38] Mahamid J Sharir A Addadi L Weiner S. Amorphous calcium phosphate is a major component of the forming fin bones of zebrafish: Indications for an amorphous precursor phase. Proc Natl Acad Sci United States America. (2008) 105:12748–53. doi: 10.1073/pnas.0803354105 PMC2529085 18753619

[39] Hempel U Reinstorf A Poppe M Fischer U Gelinsky M Pompe W. Proliferation and differentiation of osteoblasts on Biocement D modified with collagen type I and citric acid. J Biomed Mater Res Part B Appl Biomater. (2004) 71:130–43. doi: 10.1002/jbm.b.v71b:1 15368237

[40] Johnsson M Richardson CF Sallis JD Nancollas GH. Adsorption and mineralization effects of citrate and phosphocitrate on hydroxyapatite. Calcified Tissue Int. (1991) 49:134–7. doi: 10.1007/BF02565136 1655175

[41] Ruiz-Agudo E Ruiz-Agudo C Di Lorenzo F Alvarez-Lloret P Ibañez-Velasco A Rodriguez-Navarro C. Citrate stabilizes hydroxylapatite precursors: implications for bone mineralization. ACS Biomater Sci Eng. (2021) 7:2346–57. doi: 10.1021/acsbiomaterials.1c00196 PMC8479724 33973778

[42] Bradt J Mertig M Teresiak A Pompe WP. Biomimetic mineralization of collagen by combined fibril assembly and calcium phosphate formation. Chem Mater. (1999) 11:2694–701. doi: 10.1021/cm991002p

[43] Lotsari A Rajasekharan AK Halvarsson M Andersson M. Transformation of amorphous calcium phosphate to bone-like apatite. Nat Commun. (2018) 9:4170. doi: 10.1038/s41467-018-06570-x 30302020 PMC6177403

[44] Irizarry AR Yan G Zeng Q Lucchesi J Hamang MJ Ma YL. Defective enamel and bone development in sodium-dependent citrate transporter (NaCT) Slc13a5 deficient mice. PloS One. (2017) 12:e0175465. doi: 10.1371/journal.pone.0175465 28406943 PMC5391028

[45] Dirckx N Zhang Q Chu EY Tower RJ Li Z Guo S. A specialized metabolic pathway partitions citrate in hydroxyapatite to impact mineralization of bones and teeth. Proc Natl Acad Sci United States America. (2022) 119:e2212178119. doi: 10.1073/pnas.2212178119 PMC9659386 36322718

[46] Costello LC Franklin RB Reynolds MA Chellaiah M. The important role of osteoblasts and citrate production in bone formation: “Osteoblast citration” as a new concept for an old relationship. Open Bone J. (2012) 4:10.2174/1876525401204010027. doi: 10.2174/1876525401204010027 PMC3815682 24194797

[47] Fu X Li Y Huang T Yu Z Ma K Yang M. Runx2/Osterix and zinc uptake synergize to orchestrate osteogenic differentiation and citrate containing bone apatite formation. Adv Sci (Weinheim Baden-Wurttemberg Germany). (2018) 5:1700755. doi: 10.1002/advs.201700755 PMC5908346 29721422

[48] Costello LC Franklin RB. A review of the important central role of altered citrate metabolism during the process of stem cell differentiation. J Regenerative Med Tissue Eng. (2013) 2:1. doi: 10.7243/2050-1218-2-1 PMC3815687 24194979

[49] Xie X Liu M Meng Q. Angelica polysaccharide promotes proliferation and osteoblast differentiation of mesenchymal stem cells by regulation of long non-coding RNA H19: An animal study. Bone Joint Res. (2019) 8:323–32. doi: 10.1302/2046-3758.87.BJR-2018-0223.R2 PMC6691372 31463041

[50] Franklin RB Chellaiah M Zou J Reynolds MA Costello LC. Evidence that osteoblasts are specialized citrate-producing cells that provide the citrate for incorporation into the structure of bone. Open Bone J. (2014) 6:1–7. doi: 10.2174/1876525401406010001 25745519 PMC4346336

[51] Väänänen HK Laitala-Leinonen T. Osteoclast lineage and function. Arch Biochem Biophys. (2008) 473:132–8. doi: 10.1016/j.abb.2008.03.037 18424258

[52] Feng X McDonald JM. Disorders of bone remodeling. Annu Rev Pathol. (2011) 6:121–45. doi: 10.1146/annurev-pathol-011110-130203 PMC3571087 20936937

[53] Francis MJ Lees RL Trujillo E Martín-Vasallo P Heersche JN Mobasheri A. ATPase pumps in osteoclasts and osteoblasts. Int J Biochem Cell Biol. (2002) 34:459–76. doi: 10.1016/S1357-2725(01)00142-X 11906818

[54] Blair HC. How the osteoclast degrades bone. BioEssays: News Rev Mol Cell Dev Biol. (1998) 20:837–46. doi: 10.1002/(SICI)1521-1878(199810)20:10<837::AID-BIES9>3.0.CO;2-D 9819571

[55] Boyle WJ Simonet WS Lacey DL. Osteoclast differentiation and activation. Nature. (2003) 423:337–42. doi: 10.1038/nature01658 12748652

[56] Hayman AR Cox TM. Tartrate-resistant acid phosphatase knockout mice. J Bone Mineral Res. (2003) 18:1905–7. doi: 10.1359/jbmr.2003.18.10.1905 14584904

[57] Del Fattore A Cappariello A Teti A. Genetics, pathogenesis and complications of osteopetrosis. Bone. (2008) 42:19–29. doi: 10.1016/j.bone.2007.08.029 17936098

[58] Mitić N Valizadeh M Leung EW de Jersey J Hamilton S Hume DA. Human tartrate-resistant acid phosphatase becomes an effective ATPase upon proteolytic activation. Arch Biochem Biophys. (2005) 439:154–64. doi: 10.1016/j.abb.2005.05.013 15950921

[59] Davies E Müller KH Wong WC Pickard CJ Reid DG Skepper JN. Citrate bridges between mineral platelets in bone. Proc Natl Acad Sci United States America. (2014) 111:E1354–1363. doi: 10.1073/pnas.1315080111 PMC3986129 24706850

[60] Xiong J Onal M Jilka RL Weinstein RS Manolagas SC O’Brien CA. Matrix-embedded cells control osteoclast formation. Nat Med. (2011) 17:1235–41. doi: 10.1038/nm.2448 PMC3192296 21909103

[61] Miyazaki T Iwasawa M Nakashima T Mori S Shigemoto K Nakamura H. Intracellular and extracellular ATP coordinately regulate the inverse correlation between osteoclast survival and bone resorption. J Biol Chem. (2012) 287:37808–23. doi: 10.1074/jbc.M112.385369 PMC3488055 22988253

[62] Matsumoto T Nagase Y Hirose J Tokuyama N Yasui T Kadono Y. Regulation of bone resorption and sealing zone formation in osteoclasts occurs through protein kinase B-mediated microtubule stabilization. J Bone Mineral Res. (2013) 28:1191–202. doi: 10.1002/jbmr.1844 23239117

[63] Yang Y Tong M Bai X Liu X Cai X Luo X. Comprehensive proteomic analysis of lysine acetylation in the foodborne pathogen trichinella spiralis. Front Microbiol. (2017) 8:2674. doi: 10.3389/fmicb.2017.02674 29375535 PMC5768625

[64] Lin PI Tai YT Chan WP Lin YL Liao MH Chen RM. Estrogen/ERα signaling axis participates in osteoblast maturation via upregulating chromosomal and mitochondrial complex gene expressions. Oncotarget. (2018) 9:1169–86. doi: 10.18632/oncotarget.23453 PMC5787428 29416685

[65] Wu GJ Cherng YG Chen JT Chang CC Liu SH Chen RM. Genistein triggers translocation of estrogen receptor-alpha in mitochondria to induce expressions of ATP synthesis-associated genes and improves energy production and osteoblast maturation. Am J Chin Med. (2021) 49:901–23. doi: 10.1142/S0192415X21500439 33853499

[66] Guntur AR Gerencser AA Le PT DeMambro VE Bornstein SA Mookerjee SA. Osteoblast-like MC3T3-E1 cells prefer glycolysis for ATP production but adipocyte-like 3T3-L1 cells prefer oxidative phosphorylation. J Bone Mineral Res. (2018) 33:1052–65. doi: 10.1002/jbmr.3390 PMC6002892 29342317

[67] Li F Li J Li S Guo S Li P. Modulatory effects of Chinese herbal medicines on energy metabolism in ischemic heart diseases. Front Pharmacol. (2020) 11:995. doi: 10.3389/fphar.2020.00995 32719602 PMC7348053

[68] Forni MF Peloggia J Trudeau K Shirihai O Kowaltowski AJ. Murine mesenchymal stem cell commitment to differentiation is regulated by mitochondrial dynamics. Stem Cells (Dayton Ohio). (2016) 34:743–55. doi: 10.1002/stem.2248 PMC4803524 26638184

[69] Ma C Tian X Kim JP Xie D Ao X Shan D. Citrate-based materials fuel human stem cells by metabonegenic regulation. Proc Natl Acad Sci United States America. (2018) 115:E11741–e11750. doi: 10.1073/pnas.1813000115 PMC6294936 30478052

[70] Wang C Liu D Zhang C Sun J Feng W Liang XJ. Defect-related luminescent hydroxyapatite-enhanced osteogenic differentiation of bone mesenchymal stem cells via an ATP-induced cAMP/PKA pathway. ACS Appl Mater Interfaces. (2016) 8:11262–71. doi: 10.1021/acsami.6b01103 27088570

[71] Nakano Y Addison WN Kaartinen MT. ATP-mediated mineralization of MC3T3-E1 osteoblast cultures. Bone. (2007) 41:549–61. doi: 10.1016/j.bone.2007.06.011 17669706

[72] Mantila Roosa SM Liu Y Turner CH. Gene expression patterns in bone following mechanical loading. J Bone Mineral Res. (2011) 26:100–12. doi: 10.1002/jbmr.193 PMC3179310 20658561

[73] Ma Y Wu Y Xia Z Li J Li X Xu P. Anti-hypoxic molecular mechanisms of rhodiola crenulata extract in zebrafish as revealed by metabonomics. Front Pharmacol. (2019) 10:1356. doi: 10.3389/fphar.2019.01356 31780949 PMC6861209

[74] Roch PJ Henkies D Carstens JC Krischek C Lehmann W Komrakova M. Ostarine and ligandrol improve muscle tissue in an ovariectomized rat model. Front Endocrinol. (2020) 11:556581. doi: 10.3389/fendo.2020.556581 PMC7528560 33042018

[75] Da W Jiang W Tao L. ROS/MMP-9 mediated CS degradation in BMSC inhibits citric acid metabolism participating in the dual regulation of bone remodelling. Cell Death Discovery. (2024) 10:77. doi: 10.1038/s41420-024-01835-5 38355572 PMC10866869

[76] Costello LC Liu Y Franklin RB Kennedy MC. Zinc inhibition of mitochondrial aconitase and its importance in citrate metabolism of prostate epithelial cells. J Biol Chem. (1997) 272:28875–81. doi: 10.1074/jbc.272.46.28875 9360955

[77] Hipps D Dobson PF Warren C McDonald D Fuller A Filby A. Detecting respiratory chain defects in osteoblasts from osteoarthritic patients using imaging mass cytometry. Bone. (2022) 158:116371. doi: 10.1016/j.bone.2022.116371 35192969

[78] Li Y Fu G Gong Y Li B Li W Liu D. BMP-2 promotes osteogenic differentiation of mesenchymal stem cells by enhancing mitochondrial activity. J Musculoskeletal Neuronal Interact. (2022) 22:123–31. PMC8919656 35234167

[79] Kim JM Jeong D Kang HK Jung SY Kang SS Min BM. Osteoclast precursors display dynamic metabolic shifts toward accelerated glucose metabolism at an early stage of RANKL-stimulated osteoclast differentiation. Cell Physiol Biochem. (2007) 20:935–46. doi: 10.1159/000110454 17982276

[80] Czupalla C Mansukoski H Pursche T Krause E Hoflack B. Comparative study of protein and mRNA expression during osteoclastogenesis. Proteomics. (2005) 5:3868–75. doi: 10.1002/pmic.200402059 16145714

[81] Fong JE Le Nihouannen D Tiedemann K Sadvakassova G Barralet JE Komarova SV. Moderate excess of pyruvate augments osteoclastogenesis. Biol Open. (2013) 2:387–95. doi: 10.1242/bio.20133269 PMC3625867 23616923

[82] Morciano P Di Giorgio ML Porrazzo A Licursi V Negri R Rong Y. Depletion of ATP-citrate lyase (ATPCL) affects chromosome integrity without altering histone acetylation in drosophila mitotic cells. Front Physiol. (2019) 10:383. doi: 10.3389/fphys.2019.00383 31019471 PMC6458238

[83] Petillo A Abruzzese V Koshal P Ostuni A Bisaccia F. Extracellular citrate is a trojan horse for cancer cells. Front Mol Biosci. (2020) 7:593866. doi: 10.3389/fmolb.2020.593866 33282912 PMC7688668

[84] Baroni MD Colombo S Libens O Pallavi R Giorgio M Martegani E. In S. cerevisiae hydroxycitric acid antagonizes chronological aging and apoptosis regardless of citrate lyase. Apoptosis: an Int J Programmed Cell Death. (2020) 25:686–96. doi: 10.1007/s10495-020-01625-1 PMC7527365 32666259

[85] Lee JV Shah SA Wellen KE. Obesity, cancer, and acetyl-CoA metabolism. Drug Discovery Today Dis Mech. (2013) 10:e55–61. doi: 10.1016/j.ddmec.2013.03.005 PMC3713850 23878588

[86] Verdone L Agricola E Caserta M Di Mauro E. Histone acetylation in gene regulation. Briefings Funct Genomics Proteomics. (2006) 5:209–21. doi: 10.1093/bfgp/ell028 16877467

[87] Tessarz P Kouzarides T. Histone core modifications regulating nucleosome structure and dynamics. Nat Rev Mol Cell Biol. (2014) 15:703–8. doi: 10.1038/nrm3890 25315270

[88] Zhang P Liu Y Jin C Zhang M Lv L Zhang X. Histone H3K9 acetyltransferase PCAF is essential for osteogenic differentiation through bone morphogenetic protein signaling and may be involved in osteoporosis. Stem Cells (Dayton Ohio). (2016) 34:2332–41. doi: 10.1002/stem.2424 27300495

[89] Krishnan RH Sadu L Das UR Satishkumar S Pranav Adithya S Saranya I. Role of p300, a histone acetyltransferase enzyme, in osteoblast differentiation. Differentiation; Res Biol Diversity. (2022) 124:43–51. doi: 10.1016/j.diff.2022.02.002 35180610

[90] Ma C Gao J Liang J Dai W Wang Z Xia M. HDAC6 inactivates Runx2 promoter to block osteogenesis of bone marrow stromal cells in age-related bone loss of mice. Stem Cell Res Ther. (2021) 12:484. doi: 10.1186/s13287-021-02545-w 34454588 PMC8403388

[91] Zhu X Yu J Du J Zhong G Qiao L Lin J. LncRNA HOXA-AS2 positively regulates osteogenesis of mesenchymal stem cells through inactivating NF-κB signalling. J Cell Mol Med. (2019) 23:1325–32. doi: 10.1111/jcmm.2019.23.issue-2 PMC6349193 30536618

[92] Dou C Li N Ding N Liu C Yang X Kang F. HDAC2 regulates FoxO1 during RANKL-induced osteoclastogenesis. Am J Physiol Cell Physiol. (2016) 310:C780–787. doi: 10.1152/ajpcell.00351.2015 26962001

[93] Li J Liu C Li Y Zheng Q Xu Y Liu B. TMCO1-mediated Ca(2+) leak underlies osteoblast functions via CaMKII signaling. Nat Commun. (2019) 10:1589. doi: 10.1038/s41467-019-09653-5 30962442 PMC6453895

[94] Kang JY Kang N Yang YM Hong JH Shin DM. The role of ca(2+)-NFATc1 signaling and its modulation on osteoclastogenesis. Int J Mol Sci. (2020) 21:3646. doi: 10.3390/ijms21103646 32455661 PMC7279283

[95] Guo Q Kang H Wang J Dong Y Peng R Zhao H. Inhibition of ACLY leads to suppression of osteoclast differentiation and function via regulation of histone acetylation. J Bone Mineral Res. (2021) 36:2065–80. doi: 10.1002/jbmr.4399 34155695

[96] Zhou L Song H Zhang Y Ren Z Li M Fu Q. Polyphyllin VII attenuated RANKL-induced osteoclast differentiation via inhibiting of TRAF6/c-Src/PI3K pathway and ROS production. BMC Musculoskeletal Disord. (2020) 21:112. doi: 10.1186/s12891-020-3077-z PMC7031869 32075617

[97] Nozik-Grayck E Woods C Stearman RS Venkataraman S Ferguson BS Swain K. Histone deacetylation contributes to low extracellular superoxide dismutase expression in human idiopathic pulmonary arterial hypertension. Am J Physiol Lung Cell Mol Physiol. (2016) 311:L124–134. doi: 10.1152/ajplung.00263.2015 PMC4967185 27233998

[98] Tokarz P Kaarniranta K Blasiak J. Inhibition of DNA methyltransferase or histone deacetylase protects retinal pigment epithelial cells from DNA damage induced by oxidative stress by the stimulation of antioxidant enzymes. Eur J Pharmacol. (2016) 776:167–75. doi: 10.1016/j.ejphar.2016.02.049 26899469

[99] Lee YH Lee NH Bhattarai G Oh YT Yu MK Yoo ID. Enhancement of osteoblast biocompatibility on titanium surface with Terrein treatment. Cell Biochem Funct. (2010) 28:678–85. doi: 10.1002/cbf.v28:8 21104936

[100] Jiang Y Luo W Wang B Wang X Gong P Xiong Y. Resveratrol promotes osteogenesis via activating SIRT1/FoxO1 pathway in osteoporosis mice. Life Sci. (2020) 246:117422. doi: 10.1016/j.lfs.2020.117422 32057903

[101] Araújo AA Pereira A Medeiros C Brito GAC Leitão RFC Araújo LS. Effects of metformin on inflammation, oxidative stress, and bone loss in a rat model of periodontitis. PloS One. (2017) 12:e0183506. doi: 10.1371/journal.pone.0183506 28847008 PMC5573680

[102] Tsuruoka S Schwartz GJ Ioka T Yamamoto H Ando H Fujimura A. Citrate reverses cyclosporin A-induced metabolic acidosis and bone resorption in rats. Am J Nephrol. (2005) 25:233–9. doi: 10.1159/000085969 15914972

[103] Cain CJ Valencia JT Ho S Jordan K Mattingly A Morales BM. Increased gs signaling in osteoblasts reduces bone marrow and whole-body adiposity in male mice. Endocrinology. (2016) 157:1481–94. doi: 10.1210/en.2015-1867 PMC4816728 26901092

[104] Vander Heiden MG Cantley LC Thompson CB. Understanding the Warburg effect: the metabolic requirements of cell proliferation. Sci (New York NY). (2009) 324:1029–33. doi: 10.1126/science.1160809 PMC2849637 19460998

[105] Zechner R Zimmermann R Eichmann TO Kohlwein SD Haemmerle G Lass A. FAT SIGNALS–lipases and lipolysis in lipid metabolism and signaling. Cell Metab. (2012) 15:279–91. doi: 10.1016/j.cmet.2011.12.018 PMC3314979 22405066

[106] Kurmi K Haigis MC. Nitrogen metabolism in cancer and immunity. Trends Cell Biol. (2020) 30:408–24. doi: 10.1016/j.tcb.2020.02.005 PMC7386658 32302552

[107] Duarte AI Santos MS Oliveira CR Moreira PI. Brain insulin signalling, glucose metabolism and females’ reproductive aging: A dangerous triad in Alzheimer’s disease. Neuropharmacology. (2018) 136:223–42. doi: 10.1016/j.neuropharm.2018.01.044 29471055

[108] Min W Fang P Huang G Shi M Zhang Z. The decline of whole-body glucose metabolism in ovariectomized rats. Exp Gerontol. (2018) 113:106–12. doi: 10.1016/j.exger.2018.09.027 30292771

[109] Bak LK Walls AB Schousboe A Waagepetersen HS. Astrocytic glycogen metabolism in the healthy and diseased brain. J Biol Chem. (2018) 293:7108–16. doi: 10.1074/jbc.R117.803239 PMC5950001 29572349

[110] Gilmour PS O’Shea PJ Fagura M Pilling JE Sanganee H Wada H. Human stem cell osteoblastogenesis mediated by novel glycogen synthase kinase 3 inhibitors induces bone formation and a unique bone turnover biomarker profile in rats. Toxicol Appl Pharmacol. (2013) 272:399–407. doi: 10.1016/j.taap.2013.07.001 23872097

[111] Li Z Frey JL Wong GW Faugere MC Wolfgang MJ Kim JK. Glucose transporter-4 facilitates insulin-stimulated glucose uptake in osteoblasts. Endocrinology. (2016) 157:4094–103. doi: 10.1210/en.2016-1583 PMC5086531 27689415

[112] Karner CM Long F. Glucose metabolism in bone. Bone. (2018) 115:2–7. doi: 10.1016/j.bone.2017.08.008 28843700 PMC6030501

[113] Liu JM Rosen CJ Ducy P Kousteni S Karsenty G. Regulation of glucose handling by the skeleton: insights from mouse and human studies. Diabetes. (2016) 65:3225–32. doi: 10.2337/db16-0053 PMC5860442 27959858

[114] Wei J Shimazu J Makinistoglu MP Maurizi A Kajimura D Zong H. Glucose uptake and runx2 synergize to orchestrate osteoblast differentiation and bone formation. Cell. (2015) 161:1576–91. doi: 10.1016/j.cell.2015.05.029 PMC4475280 26091038

[115] Xiao Y Li B Liu J. MicroRNA−148a inhibition protects against ovariectomy−induced osteoporosis through PI3K/AKT signaling by estrogen receptor α. Mol Med Rep. (2018) 17:7789–96. doi: 10.3892/mmr.2018.8845 29620276

[116] Campello RS Fátima LA Barreto-Andrade JN Lucas TF Mori RC Porto CS. Estradiol-induced regulation of GLUT4 in 3T3-L1 cells: involvement of ESR1 and AKT activation. J Mol Endocrinol. (2017) 59:257–68. doi: 10.1530/JME-17-0041 28729437

[117] Cookman CJ Belcher SM. Estrogen receptor-β Up-regulates IGF1R expression and activity to inhibit apoptosis and increase growth of medulloblastoma. Endocrinology. (2015) 156:2395–408. doi: 10.1210/en.2015-1141 PMC4475721 25885794

[118] Alekos NS Moorer MC Riddle RC. Dual effects of lipid metabolism on osteoblast function. Front Endocrinol. (2020) 11:578194. doi: 10.3389/fendo.2020.578194 PMC7538543 33071983

[119] Chen JS Sambrook PN. Antiresorptive therapies for osteoporosis: a clinical overview. Nat Rev Endocrinol. (2011) 8:81–91. doi: 10.1038/nrendo.2011.146 21894214

[120] Adamek G Felix R Guenther HL Fleisch H. Fatty acid oxidation in bone tissue and bone cells in culture. Characterization and hormonal influences. Biochem J. (1987) 248:129–37. doi: 10.1042/bj2480129 PMC1148509 3325035

[121] Kevorkova O Martineau C Martin-Falstrault L Sanchez-Dardon J Brissette L Moreau R. Low-bone-mass phenotype of deficient mice for the cluster of differentiation 36 (CD36). PloS One. (2013) 8:e77701. doi: 10.1371/journal.pone.0077701 24204923 PMC3808405

[122] Kim SP Li Z Zoch ML Frey JL Bowman CE Kushwaha P. : Fatty acid oxidation by the osteoblast is required for normal bone acquisition in a sex- and diet-dependent manner. JCI Insight. (2017) 2:e92704. doi: 10.1172/jci.insight.92704 28814665 PMC5621897

[123] Frey JL Li Z Ellis JM Zhang Q Farber CR Aja S. Wnt-Lrp5 signaling regulates fatty acid metabolism in the osteoblast. Mol Cell Biol. (2015) 35:1979–91. doi: 10.1128/MCB.01343-14 PMC4420919 25802278

[124] Sharma D Yu Y Shen L Zhang GF Karner CM. SLC1A5 provides glutamine and asparagine necessary for bone development in mice. eLife. (2021) 10:e71595. doi: 10.7554/eLife.71595.sa2 34647520 PMC8553342

[125] Yu Y Newman H Shen L Sharma D Hu G Mirando AJ. : glutamine metabolism regulates proliferation and lineage allocation in skeletal stem cells. Cell Metab. (2019) 29:966–978.e964. doi: 10.1016/j.cmet.2019.01.016 30773468 PMC7062112

[126] Stegen S Devignes CS Torrekens S Van Looveren R Carmeliet P Carmeliet G. Glutamine metabolism in osteoprogenitors is required for bone mass accrual and PTH-induced bone anabolism in male mice. J Bone Mineral Res. (2021) 36:604–16. doi: 10.1002/jbmr.4219 33253422

[127] Huang T Liu R Fu X Yao D Yang M Liu Q. Aging reduces an ERRalpha-directed mitochondrial glutaminase expression suppressing glutamine anaplerosis and osteogenic differentiation of mesenchymal stem cells. Stem Cells (Dayton Ohio). (2017) 35:411–24. doi: 10.1002/stem.2470 27501743

[128] Singh K Krug L Basu A Meyer P Treiber N Vander Beken S. Alpha-Ketoglutarate curbs differentiation and induces cell death in mesenchymal stromal precursors with mitochondrial dysfunction. Stem Cells (Dayton Ohio). (2017) 35:1704–18. doi: 10.1002/stem.2629 28398002

[129] Karner CM Esen E Okunade AL Patterson BW Long F. Increased glutamine catabolism mediates bone anabolism in response to WNT signaling. J Clin Invest. (2015) 125:551–62. doi: 10.1172/JCI78470 PMC4319407 25562323

[130] Harrison AP Tygesen MP Sawa-Wojtanowicz B Husted S Tatara MR. Alpha-ketoglutarate treatment early in postnatal life improves bone density in lambs at slaughter. Bone. (2004) 35:204–9. doi: 10.1016/j.bone.2004.03.016 15207758

[131] Filip RS Pierzynowski SG Lindegard B Wernerman J Haratym-Maj A Podgurniak M. Alpha-ketoglutarate decreases serum levels of C-terminal cross-linking telopeptide of type I collagen (CTX) in postmenopausal women with osteopenia: six-month study. Int J Vit Nutr Res Internationale Z fur Vitamin- und Ernahrungsforschung J Int Vitaminol Nutr. (2007) 77:89–97. doi: 10.1024/0300-9831.77.2.89 17896582

[132] Radzki RP Bienko M Pierzynowski SG. Anti-osteopenic effect of alpha-ketoglutarate sodium salt in ovariectomized rats. J Bone Mineral Metab. (2012) 30:651–9. doi: 10.1007/s00774-012-0377-x 22864414

[133] Tatara Pierzynowski SG Majcher P Krupski W Brodzki A. Studziński T: Effect of alpha-ketoglutarate (AKG) on mineralisation, morphology and mechanical endurance of femur and tibia in Turkey. Bull Vet Inst Pulawy. (2004) 48:305–9.

[134] Lee S Kim HS Kim MJ Min KY Choi WS You JS. Glutamine metabolite α-ketoglutarate acts as an epigenetic co-factor to interfere with osteoclast differentiation. Bone. (2021) 145:115836. doi: 10.1016/j.bone.2020.115836 33383217

[135] Watson JA Lowenstein JM. Citrate and the conversion of carbohydrate into fat. Fatty acid synthesis by a combination of cytoplasm and mitochondria. J Biol Chem. (1970) 245:5993–6002. doi: 10.1016/S0021-9258(18)62653-5 5484459

[136] Mycielska ME Dettmer K Rümmele P Schmidt K Prehn C Milenkovic VM. Extracellular citrate affects critical elements of cancer cell metabolism and supports cancer development *in vivo* . Cancer Res. (2018) 78:2513–23. doi: 10.1158/0008-5472.CAN-17-2959 29510993

[137] Zhang W Chen H Ding Y Xiang Q Zhao J Feng W. Effect of chromium citrate on the mechanism of glucose transport and insulin resistance in Buffalo rat liver cells. Indian J Pharmacol. (2020) 52:31–8. doi: 10.4103/ijp.IJP_608_18 PMC7074430 32201444

[138] Chen Y Du J Zhao YT Zhang L Lv G Zhuang S. Histone deacetylase (HDAC) inhibition improves myocardial function and prevents cardiac remodeling in diabetic mice. Cardiovasc Diabetol. (2015) 14:99. doi: 10.1186/s12933-015-0262-8 26245924 PMC4527099

[139] Li AM He B Karagiannis D Li Y Jiang H Srinivasan P. Serine starvation silences estrogen receptor signaling through histone hypoacetylation. Proc Natl Acad Sci United States America. (2023) 120:e2302489120. doi: 10.1073/pnas.2302489120 PMC10515173 37695911

[140] Giudetti AM Stanca E Siculella L Gnoni GV Damiano F. Nutritional and hormonal regulation of citrate and carnitine/acylcarnitine transporters: two mitochondrial carriers involved in fatty acid metabolism. Int J Mol Sci. (2016) 17:817. doi: 10.3390/ijms17060817 27231907 PMC4926351

[141] Iacobazzi V Infantino V Palmieri F. Transcriptional regulation of the mitochondrial citrate and carnitine/acylcarnitine transporters: two genes involved in fatty acid biosynthesis and β-oxidation. Biology. (2013) 2:284–303. doi: 10.3390/biology2010284 24832661 PMC4009865

[142] Jiang L Boufersaoui A Yang C Ko B Rakheja D Guevara G. Quantitative metabolic flux analysis reveals an unconventional pathway of fatty acid synthesis in cancer cells deficient for the mitochondrial citrate transport protein. Metab Eng. (2017) 43:198–207. doi: 10.1016/j.ymben.2016.11.004 27856334 PMC5429990

[143] Lundby A Lage K Weinert BT Bekker-Jensen DB Secher A Skovgaard T. Proteomic analysis of lysine acetylation sites in rat tissues reveals organ specificity and subcellular patterns. Cell Rep. (2012) 2:419–31. doi: 10.1016/j.celrep.2012.07.006 PMC4103158 22902405

[144] Kuda O Pietka TA Demianova Z Kudova E Cvacka J Kopecky J. Sulfo-N-succinimidyl oleate (SSO) inhibits fatty acid uptake and signaling for intracellular calcium via binding CD36 lysine 164: SSO also inhibits oxidized low density lipoprotein uptake by macrophages. J Biol Chem. (2013) 288:15547–55. doi: 10.1074/jbc.M113.473298 PMC3668716 23603908

[145] Abo Alrob O Lopaschuk GD. Role of CoA and acetyl-CoA in regulating cardiac fatty acid and glucose oxidation. Biochem Soc Trans. (2014) 42:1043–51. doi: 10.1042/BST20140094 25110000

[146] Li Y Li YC Liu XT Zhang L Chen YH Zhao Q. Blockage of citrate export prevents TCA cycle fragmentation via Irg1 inactivation. Cell Rep. (2022) 38:110391. doi: 10.1016/j.celrep.2022.110391 35172156

[147] Kasprzyk-Pawelec A Tan M Phua YL Rahhal R McIntosh A Fernandez H. Loss of the mitochondrial citrate carrier, Slc25a1/CIC disrupts embryogenesis via 2-Hydroxyglutarate. bioRxiv: preprint Server Biol. (2023) 31:2023.07.18.549409. doi: 10.1101/2023.07.18.549409

[148] Kumar A Cordes T Thalacker-Mercer AE Pajor AM Murphy AN Metallo CM. NaCT/SLC13A5 facilitates citrate import and metabolism under nutrient-limited conditions. Cell Rep. (2021) 36:109701. doi: 10.1016/j.celrep.2021.109701 34525352 PMC8500708

[149] Łuczkowska K Rogińska D Kulig P Bielikowicz A Baumert B Machaliński B. Bortezomib-induced epigenetic alterations in nerve cells: focus on the mechanisms contributing to the peripheral neuropathy development. Int J Mol Sci. (2022) 23:2431. doi: 10.3390/ijms23052431 35269574 PMC8910765

[150] Wang J Zhang K Chen X Liu X Teng H Zhao M. Epigenetic activation of ASCT2 in the hippocampus contributes to depression-like behavior by regulating D-serine in mice. Front Mol Neurosci. (2017) 10:139. doi: 10.3389/fnmol.2017.00139 28536503 PMC5422558

[151] Weitzmann MN Ofotokun I. Physiological and pathophysiological bone turnover - role of the immune system. Nat Rev Endocrinol. (2016) 12:518–32. doi: 10.1038/nrendo.2016.91 PMC5857945 27312863

[152] Weitzmann MN Pacifici R. Estrogen deficiency and bone loss: an inflammatory tale. J Clin Invest. (2006) 116:1186–94. doi: 10.1172/JCI28550 PMC1451218 16670759

[153] Lorenzo J. Cytokines and bone: osteoimmunology. Handb Exp Pharmacol. (2020) 262:177–230. doi: 10.1007/164_2019_346 32006259

[154] Cohen-Solal ME Graulet AM Denne MA Gueris J Baylink D de Vernejoul MC. Peripheral monocyte culture supernatants of menopausal women can induce bone resorption: involvement of cytokines. J Clin Endocrinol Metab. (1993) 77:1648–53. doi: 10.1210/jcem.77.6.8263153 8263153

[155] Zheng SX Vrindts Y Lopez M De Groote D Zangerle PF Collette J. Increase in cytokine production (IL-1 beta, IL-6, TNF-alpha but not IFN-gamma, GM-CSF or LIF) by stimulated whole blood cells in postmenopausal osteoporosis. Maturitas. (1997) 26:63–71. doi: 10.1016/S0378-5122(96)01080-8 9032749

[156] Abildgaard J Tingstedt J Zhao Y Hartling HJ Pedersen AT Lindegaard B. Increased systemic inflammation and altered distribution of T-cell subsets in postmenopausal women. PloS One. (2020) 15:e0235174. doi: 10.1371/journal.pone.0235174 32574226 PMC7310708

[157] Zupan J Komadina R Marc J. The relationship between osteoclastogenic and anti-osteoclastogenic pro-inflammatory cytokines differs in human osteoporotic and osteoarthritic bone tissues. J Biomed Sci. (2012) 19:28. doi: 10.1186/1423-0127-19-28 22380539 PMC3307025

[158] Pacifici R Rifas L McCracken R Vered I McMurtry C Avioli LV. Ovarian steroid treatment blocks a postmenopausal increase in blood monocyte interleukin 1 release. Proc Natl Acad Sci United States America. (1989) 86:2398–402. doi: 10.1073/pnas.86.7.2398 PMC286920 2522659

[159] Romas E Martin TJ. Cytokines in the pathogenesis of osteoporosis. Osteoporosis Int. (1997) 7 Suppl 3:S47–53. doi: 10.1007/BF03194342 9536302

[160] Pacifici R. Estrogen, cytokines, and pathogenesis of postmenopausal osteoporosis. J Bone Mineral Res. (1996) 11:1043–51. doi: 10.1002/jbmr.5650110802 8854239

[161] Lei Z Xiaoying Z Xingguo L. Ovariectomy-associated changes in bone mineral density and bone marrow haematopoiesis in rats. Int J Exp Pathol. (2009) 90:512–9. doi: 10.1111/j.1365-2613.2009.00661.x PMC2768149 19765105

[162] Garlanda C Dinarello CA Mantovani A. The interleukin-1 family: back to the future. Immunity. (2013) 39:1003–18. doi: 10.1016/j.immuni.2013.11.010 PMC3933951 24332029

[163] Tseng HW Samuel SG Schroder K Lأ©vesque JP Alexander KA. Inflammasomes and the IL-1 family in bone homeostasis and disease. Curr Osteoporosis Rep. (2022) 20:170–85. doi: 10.1007/s11914-022-00729-8 PMC9209354 35567665

[164] Zhang Y Shen X Cheng L Chen R Zhao F Zhong S. Toll-like receptor 4 knockout protects against diabetic-induced imbalance of bone metabolism via autophagic suppression. Mol Immunol. (2020) 117:12–9. doi: 10.1016/j.molimm.2019.10.025 31731054

[165] Kishimoto T Akira S Narazaki M Taga T. Interleukin-6 family of cytokines and gp130. Blood. (1995) 86:1243–54. doi: 10.1182/blood.V86.4.1243.bloodjournal8641243 7632928

[166] Malaval L Aubin JE. Biphasic effects of leukemia inhibitory factor on osteoblastic differentiation. J Cell Biochem Supplement. (2001) Suppl 36:63–70. doi: 10.1002/jcb.1086 11455571

[167] Malaval L Liu F Vernallis AB Aubin JE. GP130/OSMR is the only LIF/IL-6 family receptor complex to promote osteoblast differentiation of calvaria progenitors. J Cell Physiol. (2005) 204:585–93. doi: 10.1002/jcp.v204:2 15751050

[168] Falconi D Aubin JE. LIF inhibits osteoblast differentiation at least in part by regulation of HAS2 and its product hyaluronan. J Bone Mineral Res. (2007) 22:1289–300. doi: 10.1359/jbmr.070417 17451373

[169] Wang T He C Yu X. Pro-inflammatory cytokines: new potential therapeutic targets for obesity-related bone disorders. Curr Drug Targets. (2017) 18:1664–75. doi: 10.2174/1389450118666170104153512 28056748

[170] Huang H Zhao N Xu X Xu Y Li S Zhang J. Dose-specific effects of tumor necrosis factor alpha on osteogenic differentiation of mesenchymal stem cells. Cell Proliferation. (2011) 44:420–7. doi: 10.1111/j.1365-2184.2011.00769.x PMC6495272 21951285

[171] Glass GE Chan JK Freidin A Feldmann M Horwood NJ Nanchahal J. TNF-alpha promotes fracture repair by augmenting the recruitment and differentiation of muscle-derived stromal cells. Proc Natl Acad Sci United States America. (2011) 108:1585–90. doi: 10.1073/pnas.1018501108 PMC3029750 21209334

[172] Hess K Ushmorov A Fiedler J Brenner RE Wirth T. TNFalpha promotes osteogenic differentiation of human mesenchymal stem cells by triggering the NF-kappaB signaling pathway. Bone. (2009) 45:367–76. doi: 10.1016/j.bone.2009.04.252 19414075

[173] Cho HH Shin KK Kim YJ Song JS Kim JM Bae YC. NF-kappaB activation stimulates osteogenic differentiation of mesenchymal stem cells derived from human adipose tissue by increasing TAZ expression. J Cell Physiol. (2010) 223:168–77. doi: 10.1002/jcp.v223:1 20049872

[174] Zhang YH Heulsmann A Tondravi MM Mukherjee A Abu-Amer Y. Tumor necrosis factor-alpha (TNF) stimulates RANKL-induced osteoclastogenesis via coupling of TNF type 1 receptor and RANK signaling pathways. J Biol Chem. (2001) 276:563–8. doi: 10.1074/jbc.M008198200 11032840

[175] Li P Schwarz EM O’Keefe RJ Ma L Looney RJ Ritchlin CT. Systemic tumor necrosis factor alpha mediates an increase in peripheral CD11bhigh osteoclast precursors in tumor necrosis factor alpha-transgenic mice. Arthritis Rheum. (2004) 50:265–76. doi: 10.1002/art.11419 14730625

[176] Lam J Takeshita S Barker JE Kanagawa O Ross FP Teitelbaum SL. TNF-alpha induces osteoclastogenesis by direct stimulation of macrophages exposed to permissive levels of RANK ligand. J Clin Invest. (2000) 106:1481–8. doi: 10.1172/JCI11176 PMC387259 11120755

[177] Azuma Y Kaji K Katogi R Takeshita S Kudo A. Tumor necrosis factor-alpha induces differentiation of and bone resorption by osteoclasts. J Biol Chem. (2000) 275:4858–64. doi: 10.1074/jbc.275.7.4858 10671521

[178] Leandro JG Espindola-Netto JM Vianna MC Gomez LS DeMaria TM Marinho-Carvalho MM. Exogenous citrate impairs glucose tolerance and promotes visceral adipose tissue inflammation in mice. Br J Nutr. (2016) 115:967–73. doi: 10.1017/S0007114516000027 26863933

[179] Xia Y Zhang X Bo A Sun J Li M. Sodium citrate inhibits the proliferation of human gastric adenocarcinoma epithelia cells. Oncol Lett. (2018) 15:6622–8. doi: 10.3892/ol.2018.8111 PMC5876446 29616124

[180] Baardman J Verberk SGS van der Velden S Gijbels MJJ van Roomen C Sluimer JC. Macrophage ATP citrate lyase deficiency stabilizes atherosclerotic plaques. Nat Commun. (2020) 11:6296. doi: 10.1038/s41467-020-20141-z 33293558 PMC7722882

[181] Namgaladze D Zukunft S Schnütgen F Kurrle N Fleming I Fuhrmann D. Polarization of human macrophages by interleukin-4 does not require ATP-citrate lyase. Front Immunol. (2018) 9:2858. doi: 10.3389/fimmu.2018.02858 30568658 PMC6290342

[182] Gotoh K Morisaki T Setoyama D Sasaki K Yagi M Igami K. Mitochondrial p32/C1qbp is a critical regulator of dendritic cell metabolism and maturation. Cell Rep. (2018) 25:1800–1815.e1804. doi: 10.1016/j.celrep.2018.10.057 30428349

[183] Kendrick SF O’Boyle G Mann J Zeybel M Palmer J Jones DE. Acetate, the key modulator of inflammatory responses in acute alcoholic hepatitis. Hepatol (Baltimore Md). (2010) 51:1988–97. doi: 10.1002/hep.23572 20232292

[184] Bhatt DP Rosenberger TA. Acetate treatment increases fatty acid content in LPS-stimulated BV2 microglia. Lipids. (2014) 49:621–31. doi: 10.1007/s11745-014-3911-x 24852320

[185] Si Z Zhou S Shen Z Luan F. High-throughput metabolomics discovers metabolic biomarkers and pathways to evaluating the efficacy and exploring potential mechanisms of osthole against osteoporosis based on UPLC/Q-TOF-MS coupled with multivariate data analysis. Front Pharmacol. (2020) 11:741. doi: 10.3389/fphar.2020.00741 32670052 PMC7326133

[186] Granchi D Caudarella R Ripamonti C Spinnato P Bazzocchi A Torreggiani E. Association between markers of bone loss and urinary lithogenic risk factors in osteopenic postmenopausal women. J Biol Regulators Homeostatic Agents. (2016) 30:145–51. 28002912

[187] Arrabal-Polo MA Girón-Prieto MS Cano-García Mdel C Poyatos-Andujar A Quesada-Charneco M Abad-Menor F. Retrospective review of serum and urinary lithogenic risk factors in patients with osteoporosis and osteopenia. Urology. (2015) 85:782–5. doi: 10.1016/j.urology.2015.01.019 25817102

[188] Pak CY Peterson RD Poindexter J. Prevention of spinal bone loss by potassium citrate in cases of calcium urolithiasis. J Urol. (2002) 168:31–4. doi: 10.1016/S0022-5347(05)64825-2 12050486

[189] Da W Tao L Wen K Tao Z Wang S Zhu Y. Protective role of melatonin against postmenopausal bone loss via enhancement of citrate secretion from osteoblasts. Front Pharmacol. (2020) 11:667. doi: 10.3389/fphar.2020.00667 32508637 PMC7248328

[190] Zhang RK Yan K Chen HF Zhang Y Li GJ Chen XG. Anti-osteoporotic drugs affect the pathogenesis of gut microbiota and its metabolites: a clinical study. Front Cell Infect Microbiol. (2023) 13:1091083. doi: 10.3389/fcimb.2023.1091083 37475958 PMC10354646

[191] Prezioso D Strazzullo P Lotti T Bianchi G Borghi L Caione P. Dietary treatment of urinary risk factors for renal stone formation. A review of CLU Working Group. Archivio Italiano di Urol Androl: Organo Ufficiale [di] Soc Italiana di Ecografia Urol e Nefrol. (2015) 87:105–20. doi: 10.4081/aiua.2015.2.105 26150027

[192] Domrongkitchaiporn S Stitchantrakul W Kochakarn W. Causes of hypocitraturia in recurrent calcium stone formers: focusing on urinary potassium excretion. Am J Kidney Dis. (2006) 48:546–54. doi: 10.1053/j.ajkd.2006.06.008 16997050

[193] Jehle S Zanetti A Muser J Hulter HN Krapf R. Partial neutralization of the acidogenic Western diet with potassium citrate increases bone mass in postmenopausal women with osteopenia. J Am Soc Nephrol: JASN. (2006) 17:3213–22. doi: 10.1681/ASN.2006030233 17035614

[194] Karp HJ Ketola ME Lamberg-Allardt CJ. Acute effects of calcium carbonate, calcium citrate and potassium citrate on markers of calcium and bone metabolism in young women. Br J Nutr. (2009) 102:1341–7. doi: 10.1017/S0007114509990195 19538811

[195] Macdonald HM Black AJ Aucott L Duthie G Duthie S Sandison R. Effect of potassium citrate supplementation or increased fruit and vegetable intake on bone metabolism in healthy postmenopausal women: a randomized controlled trial. Am J Clin Nutr. (2008) 88:465–74. doi: 10.1093/ajcn/88.2.465 18689384

[196] Lane SW Williams DA Watt FM. Modulating the stem cell niche for tissue regeneration. Nat Biotechnol. (2014) 32:795–803. doi: 10.1038/nbt.2978 25093887 PMC4422171

[197] Tran RT Wang L Zhang C Huang M Tang W Zhang C. Synthesis and characterization of biomimetic citrate-based biodegradable composites. J Biomed Mater Res Part A. (2014) 102:2521–32. doi: 10.1002/jbm.a.v102.8 PMC3931750 23996976

[198] Xie D Guo J Mehdizadeh M Tran RT Chen R Sun D. Development of injectable citrate-based bioadhesive bone implants. J Mater Chem B. (2015) 3:387–98. doi: 10.1039/C4TB01498G PMC4286886 25580247

[199] Schneiders W Reinstorf A Pompe W Grass R Biewener A Holch M. Effect of modification of hydroxyapatite/collagen composites with sodium citrate, phosphoserine, phosphoserine/RGD-peptide and calcium carbonate on bone remodelling. Bone. (2007) 40:1048–59. doi: 10.1016/j.bone.2006.11.019 17223400

[200] Zhao C Zeng Z Qazvini NT Yu X Zhang R Yan S. Thermoresponsive citrate-Based graphene oxide scaffold enhances bone regeneration from BMP9-Stimulated adipose-Derived mesenchymal stem cells. ACS Biomater Sci Eng. (2018) 4:2943–55. doi: 10.1021/acsbiomaterials.8b00179 PMC6425978 30906855

[201] Luo K Wang L Tang J Zeng X Chen X Zhang P. Enhanced biomineralization of shape memory composite scaffolds from citrate functionalized amorphous calcium phosphate for bone repair. J Mater Chem B. (2021) 9:9191–203. doi: 10.1039/D1TB01554K 34698324

[202] Mehdizadeh M Weng H Gyawali D Tang L Yang J. Injectable citrate-based mussel-inspired tissue bioadhesives with high wet strength for sutureless wound closure. Biomaterials. (2012) 33:7972–83. doi: 10.1016/j.biomaterials.2012.07.055 PMC3432175 22902057

[203] Guo J Kim GB Shan D Kim JP Hu J Wang W. Click chemistry improved wet adhesion strength of mussel-inspired citrate-based antimicrobial bioadhesives. Biomaterials. (2017) 112:275–86. doi: 10.1016/j.biomaterials.2016.10.010 PMC5121090 27770631

[204] Motlagh D Allen J Hoshi R Yang J Lui K Ameer G. Hemocompatibility evaluation of poly(diol citrate) *in vitro* for vascular tissue engineering. J Biomed Mater Res Part A. (2007) 82:907–16. doi: 10.1002/jbm.a.v82a:4 17335023

[205] Wu X Dai H Yu S Zhao Y Long Y Li W. Magnesium calcium phosphate cement incorporating citrate for vascularized bone regeneration. ACS Biomater Sci Eng. (2020) 6:6299–308. doi: 10.1021/acsbiomaterials.0c00929 33449642

[206] Guo J Xie Z Tran RT Xie D Jin D Bai X. Click chemistry plays a dual role in biodegradable polymer design. Adv Mater (Deerfield Beach Fla). (2014) 26:1906–11. doi: 10.1002/adma.201305162 PMC3969723 24375469

[207] Fernandes MH Alves MM Cebotarenco M Ribeiro IAC Grenho L Gomes PS. Citrate zinc hydroxyapatite nanorods with enhanced cytocompatibility and osteogenesis for bone regeneration. Mater Sci Eng C Mater Biol Appl. (2020) 115:111147. doi: 10.1016/j.msec.2020.111147 32600733

[208] Huard K Brown J Jones JC Cabral S Futatsugi K Gorgoglione M. Discovery and characterization of novel inhibitors of the sodium-coupled citrate transporter (NaCT or SLC13A5). Sci Rep. (2015) 5:17391. doi: 10.1038/srep17391 26620127 PMC4664966

[209] Huard K Gosset JR Montgomery JI Gilbert A Hayward MM Magee TV. Optimization of a dicarboxylic series for *in vivo* inhibition of citrate transport by the solute carrier 13 (SLC13) family. J Med Chem. (2016) 59:1165–75. doi: 10.1021/acs.jmedchem.5b01752 26734723

[210] Tan M Mosaoa R Graham GT Kasprzyk-Pawelec A Gadre S Parasido E. : Inhibition of the mitochondrial citrate carrier, Slc25a1, reverts steatosis, glucose intolerance, and inflammation in preclinical models of NAFLD/NASH. Cell Death Differ. (2020) 27:2143–57. doi: 10.1038/s41418-020-0491-6 PMC7308387 31959914

[211] Fernandez HR Gadre SM Tan M Graham GT Mosaoa R Ongkeko MS. The mitochondrial citrate carrier, SLC25A1, drives stemness and therapy resistance in non-small cell lung cancer. Cell Death Differ. (2018) 25:1239–58. doi: 10.1038/s41418-018-0101-z PMC6030199 29651165

[212] Tashjian AH Jr. Whedon GD. Kinetics of human citrate metabolism: studies in normal subjects and in patients with bone disease. J Clin Endocrinol Metab. (1963) 23:1029–43. doi: 10.1210/jcem-23-10-1029 14067890

[213] Xiong Y Mi BB Lin Z Hu YQ Yu L Zha KK. The role of the immune microenvironment in bone, cartilage, and soft tissue regeneration: from mechanism to therapeutic opportunity. Military Med Res. (2022) 9:65. doi: 10.1186/s40779-022-00426-8 PMC9675067 36401295

[214] Prochaska M Taylor EN Curhan G. Menopause and risk of kidney stones. J Urol. (2018) 200:823–8. doi: 10.1016/j.juro.2018.04.080 PMC6556766 29730204

[215] Haferkamp S Drexler K Federlin M Schlitt HJ Berneburg M Adamski J. Extracellular citrate fuels cancer cell metabolism and growth. Front Cell Dev Biol. (2020) 8:602476. doi: 10.3389/fcell.2020.602476 33425906 PMC7793864

